# Natural history of the social millipede *Brachycybe lecontii* Wood, 1864

**DOI:** 10.3897/BDJ.8.e50770

**Published:** 2020-04-03

**Authors:** Victoria L. Wong, Derek A. Hennen, Angie M. Macias, Michael S. Brewer, Matt T. Kasson, Paul Marek

**Affiliations:** 1 Department of Entomology, Virginia Polytechnic Institute and State University, Blacksburg, United States of America Department of Entomology, Virginia Polytechnic Institute and State University Blacksburg United States of America; 2 Division of Plant and Soil Sciences, West Virginia University, Morgantown, United States of America Division of Plant and Soil Sciences, West Virginia University Morgantown United States of America; 3 Department of Biology, East Carolina University, Greenville, United States of America Department of Biology, East Carolina University Greenville United States of America; 4 Virginia Tech, Blacksburg, United States of America Virginia Tech Blacksburg United States of America

**Keywords:** subsocial, quasi-social, fungivore, Diplopoda, Colobognatha, Platydesmida, Andrognathidae

## Abstract

The millipede *Brachycybe lecontii* Wood, 1864 is a fungivorous social millipede known for paternal care of eggs and forming multi-generational aggregations. We investigated the life history, paternal care, chemical defence, feeding and social behaviour of *B. lecontii* and provided morphological and anatomical descriptions, using light and scanning electron microscopy. Based on observations of *B. lecontii* from 13 locations throughout its distribution, we report the following natural history aspects. The oviposition period of *B. lecontii* lasted from mid-April to late June and the incubation period lasted 3–4 weeks. Only males cared for the eggs and subsequent care of juveniles was not observed. In one case, the clutches of two males became combined and they were later cared for by only one of the males. The defensive compound of *B. lecontii* is stored in large glands occupying a third of the paranotal volume and were observed only in stadia II millipedes and older. We observed *B. lecontii* feeding on fungi of the order Polyporales and describe a cuticular structure on the tip of the labrum that may relate to fungivory. We found that their stellate-shaped aggregations (pinwheels) do not form in the absence of fungus and suggest the aggregation is associated with feeding. We describe and illustrate a previously undescribed comb-like structure on the tibia and tarsi of the six anterior-most leg-pairs and measure the colour and spectral reflectance of the *B. lecontii* exoskeleton.

## Introduction

The genus *Brachycybe* Wood, 1864 (Platydesmida: Andrognathidae) consists of eight nominal species with a fragmentary distribution in Japan, South Korea, east China, Taiwan, south-eastern and south-central United States (U.S.) and the western U.S.—along the Pacific Coast and Sierra Nevada (Shelley et al. 2005, Mikhaljova et al. 2010). The genus diverged from related andrognathid taxa approximately 50 million years ago (Ma) and the divergence between Asian, eastern North American and western North American species of *Brachycybe* occurred 17–20 Ma (Brewer et al. 2012). Across its distribution, *Brachycybe* can be found in mesic temperate deciduous forests on decaying logs in groups of up to a hundred individuals. Field notes by Cope (1870), Hoffman (1950) and Shelley et al. (2005) on *Brachycybe* species described encountering them in a variety of microhabitats associated with detritus or decaying wood. They are frequently found in close proximity to fungus and are assumed to feed on the fungal tissue (Gardner 1974, Macias et al. 2019). One species, *Brachycybe producta* Loomis, 1936, was observed eating corticioid Basidiomycete fungi in the genus *Peniophora* (Gardner 1974). The fungal food sources of *B. lecontii* were recently surveyed by culturing gut contents and the millipedes were found to harbour a surprisingly diverse fungal community, spanning some 176 genera of fungi from 39 orders and four phyla (Macias et al. 2019). The fungal communities of other species of *Brachycybe* remain unstudied.

*Brachycybe* individuals are small, reaching 4–5 cm in length and up to 4 mm in width (Gardner 1974). Like all platydesmidan millipedes, they are lightly pigmented with colouration varying from beige and pale pink to red (Gardner 1974, Shelley et al. 2005). The flat, wide, keel-shaped paranota are large, comprising up to 60% of an individual's body width and contain defence glands described as slender lengthy tubes (Eisner et al. 1978). Heads of *Brachycybe* are small, roughly one-third of their body width and exhibit reduced mandibles and chewing musculature, including the apodemes (Manton 1961, Gardner 1974, Blanke and Wesener 2014). Eyes are absent, as is the case throughout the Platydesmida, and the anterior portion of the head is covered by a smooth head capsule (Gardner 1974, Shelley et al. 2005). Their dorsoventrally flattened body and closely-spaced paranota impart a feather-like appearance, explaining the colloquial name for the genus, the “feather millipedes”.

Species-level morphological identification does not include features of the gonopods as they appear largely uniform and leg-like in appearance (Gardner 1974, Brewer et al. 2012). Gonopods of platydesmidan millipedes are the ninth and tenth leg pairs that are modified as sperm transfer organs. Some gonopodal shape variation between species have been observed, but insufficient data have been collected to determine if these traits are indicative of species-level differences (Brewer et al. 2012). In addition, the small size of the gonopods makes them impractical for identification purposes without the use of high-magnification optics like scanning electron microscopy (Gardner 1974, Brewer et al. 2012). Somatic features, such as the shape and texture of the paranota and collum (the first body ring), have traditionally been used for species identification (Gardner 1974, Brewer et al. 2012). For example, the two eastern U.S. species, *B. lecontii* and *B. petasata* Loomis, 1936, can be easily differentiated in the field, based on the presence of a deep anterior notch on the collum of *B. petasata* (Gardner 1974).

An exceptional aspect of *Brachycybe* millipedes is that they display similar types of social behaviour and occur in persistent colonies of individuals with overlapping generations (Gardner 1974). Sociality is variously defined as the organisms having one or more of the following characteristics: (1) division of labour with a caste system composed of reproductive and non-reproductive members, (2) cooperation in caring for the young, (3) a shared nest or aggregation space and (4) overlapping generations (Gullan and Cranston 2014). Sociality, rare amongst millipedes, has evolved independently in the subterclass Colobognatha, with most other millipedes being solitary. Possible exceptions are maternal brood care in the polydesmidan taxa: *Eutrichodesmus* spp., *Cryptocorypha* sp., and *Gasterogramma psi* (Murakami 1972, Kudo et al. 2014, Mesibov 2003). Several platydesmidan taxa, including *Brachycybe*, are known for their gregarious behaviour in which multiple individuals arrange themselves in a stellate formation, with their heads placed together in a central hub and their bodies radiating outwards, like the spokes of a wheel (Lewis 1984, Shear 2015). These aggregations are stable and persistent, with no visible activity, unless disturbed (Lewis 1984). Colloquially referred to as “pinwheels”, these aggregations have been hypothesised to be related to defence or feeding (Lewis 1984, Dury et al. 2014, Shear 2015).

Platydesmidan millipedes display subsocial behaviour and not eusociality since they apparently lack a caste system and all individuals are likely able to reproduce. Many members of Platydesmida care for their young. Paternal care of eggs is displayed by the platydesmidan genera *Brachycybe*, *Platydesmus*, *Pseudodesmus* and *Yamasinaium* (Lewis 1984, Kudo et al. 2009, Kudo et al. 2010). In contrast, colobognath females brood the eggs in *Dolistenus* species (Platydesmida), *Orsiboe ichigomensis* (Polyzoniida) and *Polyzonium germanicum* (Polyzoniida) (Enghoff 1984). In *Brachycybe*, paternal care has been observed in five species and likely occurs in the other three species as well: *B. lecontii* and *B. petasata* (eastern U.S.), *B. producta* and *B. rosea* (western U.S.), and *B. nodulosa* (Japan and South Korea) (Gardner 1974, Murakami 1963, Tallamy 2001, Kudo et al. 2009, Kudo et al. 2010). Research on paternal care has focused primarily on *B. nodulosa* (see summary in Kudo et al. 2014), which is the Japanese sister species of *B. lecontii* (Brewer et al. 2012). In *Brachycybe nodulosa* (=*Bazillozonium nodulosum*), females lay clutches of 23–78 eggs directly into the care of the male, which then wraps his body around the eggs and holds the clutch with his legs. The eggs are then brooded by the male until hatching. Paternal care has been experimentally shown to be required for the survival of the eggs (Murakami 1962a, Murakami 1963, Kudo et al. 2010).

**Figure 1.**
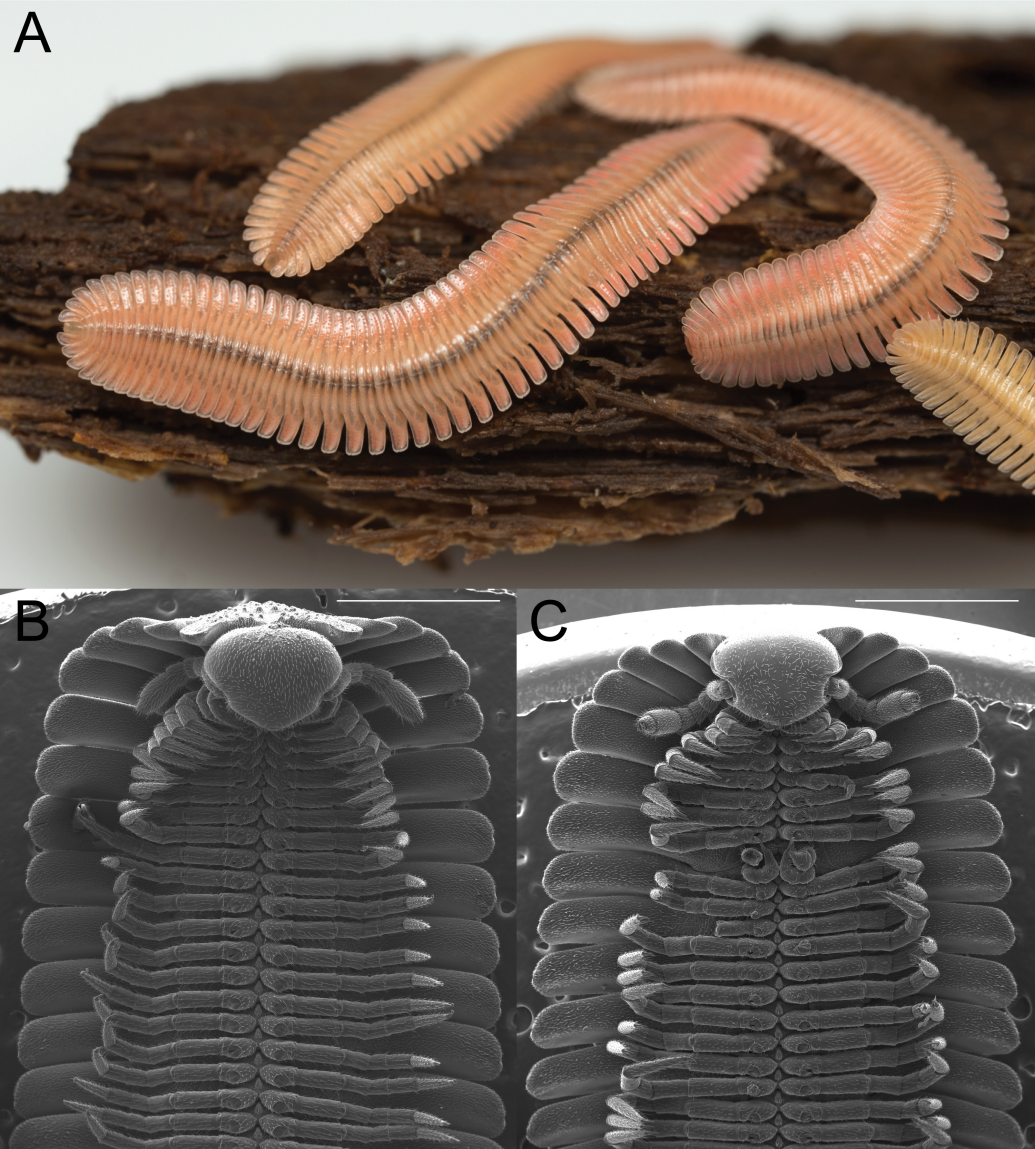
The social millipede *Brachycybe lecontii*. **A.** Several *B. lecontii* individuals atop a piece of decaying hickory wood from their habitat (Shortt Gap, Tazewell County, Virginia); **B.** Female *B. lecontii*, scanning electron micrograph (SEM) of the ventral surface of rings 1–14; **C.** Male *B. lecontii*, SEM of the ventral surface of rings 1–13. Scale bars B, C = 1 mm.

*Brachycybe lecontii* Wood, 1864 (Fig. 1) was the first andrognathid millipede described from the U.S., based on collections from Georgia (no further locality details were provided). Wood (1864) first placed *B. lecontii* in the family Siphonophoridae. However, in 1869, the family Andrognathidae was proposed by Edward Drinker Cope to contain the new species *Andrognathus corticarius* and, a year later, Cope transferred *Brachycybe* to this family (Cope 1869, Cope 1870, Shorter et al. 2018). After 1869, andrognathids were variously divided up and placed in families such as Polyzoniidae and Platydesmidae until 1928, when Cook and Loomis resurrected the family Andrognathidae and placed it in the order Platydesmida (McNeil 1888, Bollman 1893, Brölemann 1900, Cook and Loomis 1928, Gardner 1974).

**Figure 2.**
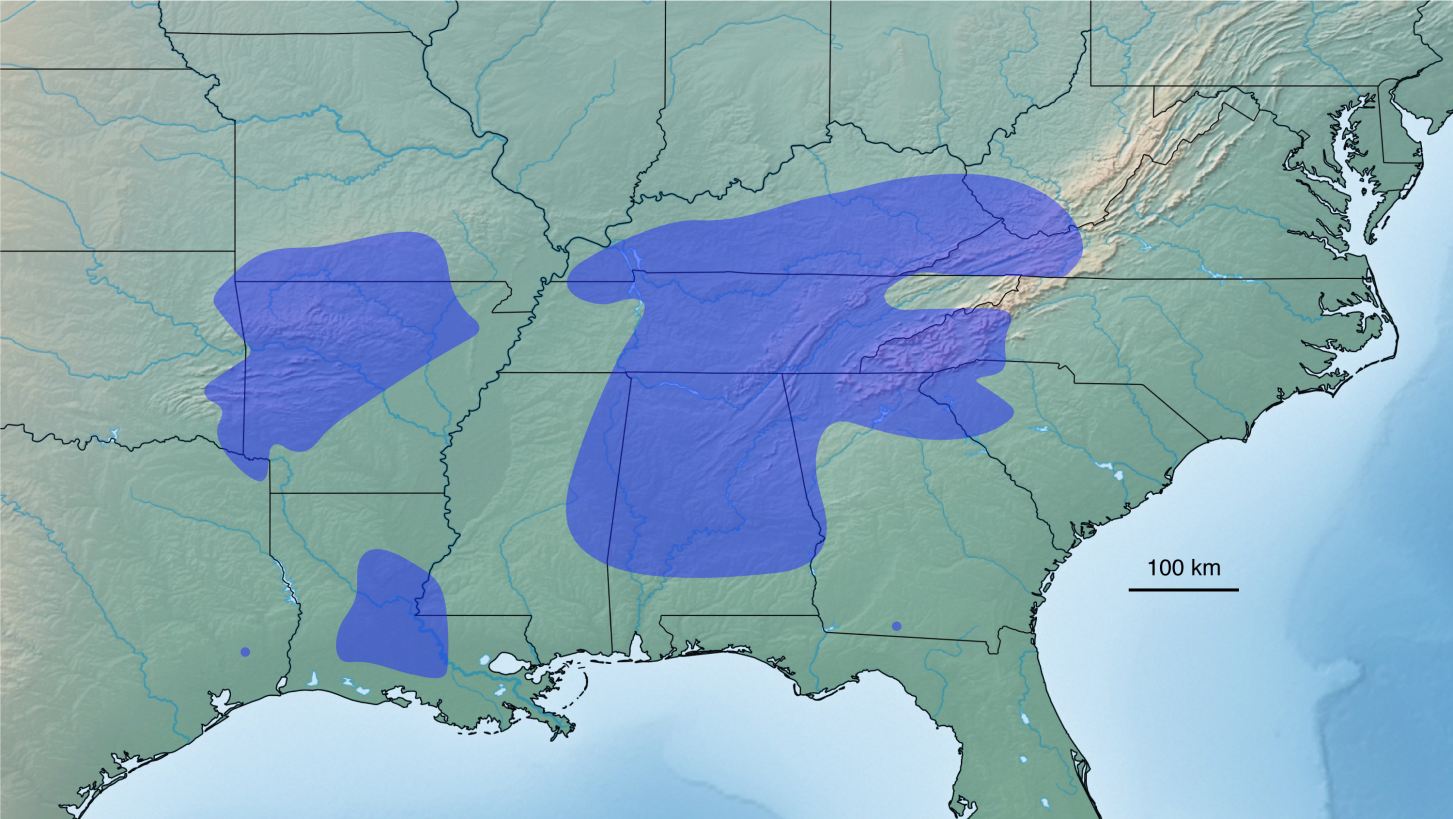
Distribution of *Brachycybe lecontii*. *Brachycybe lecontii* has a large, fragmented distribution (in blue), extending from southern Missouri and West Virginia to eastern Texas and southern Georgia.

*Brachycybe lecontii* is one of eight species in the genus, the others are *B. petasata* (Southern Appalachians), *B. picta* (coastal California), *B. producta* (California and Oregon), *B. rosea* (Sierra Nevada, California), *B. nodulosa* (Japan, South Korea), *B. disticha* (Taiwan) and *B. cooki* (China) (Shelley et al. 2005, Mikhaljova et al. 2010). Brewer et al. (2012) analysed the molecular species phylogeny of the genus and found that *B. nodulosa* is in a clade containing the U.S. species and is the sister species of *B. lecontii*. They also found that the genus *Brachycybe* may be under-split taxonomically and may contain two cryptic species in the U.S. The clade comprising *B. lecontii*, made up of 81 mtDNA haplotypes, includes four phylogenetically and geographically distinct (parapatric) clades. However, these clades are not always morphologically distinct (Brewer et al. 2012). Shelley et al. (2005) compiled available locality information on *B. lecontii* and showed that the species has a large fragmentary distribution across the eastern U.S. with five geographically separated groups (Fig. 2). Brewer et al. (2012) refrained from naming the four clades (“LC 1–4”) discovered in their analysis as species, as they indicated the clades were relatively recent in origin and lacked support from morphological characters.

The published literature on *Brachycybe* is limited to taxonomic and morphological studies and the detailed natural history work led by Murakami and Kudo on paternal care of eggs and development in the East Asian species *B. nodulosa* (Murakami 1962a, Murakami 1962b, Murakami 1963, Kudo et al. 2009, Kudo et al. 2010, Kudo et al. 2014). Youngsteadt and McAllister (2014) recorded detailed observational notes on *B. lecontii* from Arkansas and suggested that the species feeds on microorganisms that live on rotting wood; however, they did not directly observe the millipedes feeding on microorganisms or describe how this was inferred. Gardner (1974) found *B. producta* feeding upon the corticioid basidiomycete fungus *Peniophora* sp. and Macias et al. (2019) observed *B. lecontii* feeding on fungi in pure plates of fungal culture (*Phlebiopsis* sp.). Youngsteadt and McAllister also observed moulting, a process that occurred over 10 days and found that the millipedes did not construct a moulting chamber or eat their shed exoskeletons as in some other millipedes (Youngsteadt and McAllister 2014). They also observed a single adult male with a clutch of 24 eggs that hatched after 21 days (Youngsteadt and McAllister 2014).

Like many species of millipedes, *B. lecontii* produces a chemical defence compound that is stored in cuticle-lined exocrine glands located in their paranota (Eisner et al. 1978, Shear 2015). The defence glands line both sides of the body and begin on ring four. The chemical compound is structurally similar to the alkaloid buzonamine produced by the millipede *Buzonium crassipes* (Shear 2015; Jones, T., unpublished data). Buzonamine was found to repel the ant *Formica obscuripes* in an experimental anti-predator bioassay (Wood et al. 2000). Based on an ancestral character state reconstruction of chemical defences upon a phylogeny of the class Diplopoda, production of alkaloids, such as buzonamine, appears to be a derived trait and the primitive chemicals in millipedes are lower mass phenols and cresols (Rodriguez et al. 2018).

Despite its cryptic diversity and fascinating biology, little is known about millipedes in the genus *Brachycybe*. This study combines field and laboratory observations and focuses on feeding behaviour, pinwheel aggregations and chemical defences. Here, we synthesise available published data for the species *B. lecontii* and provide new observations on its life history that include descriptions of anatomy, morphology, post-embryonic development, social behaviour and aspects of parental care.

## Materials and methods

We collected *B. lecontii* specimens from 13 localities in the eastern U.S. during the spring and summer of 2015–2017 (Table 1). These localities were distributed across six states: Alabama, Arkansas, North Carolina, Missouri, Tennessee and Virginia. Millipedes were collected in deciduous forests by examining the undersides of logs or by raking leaf litter and collecting specimens by hand, according to methods outlined by Means et al. (2015). Specimens were stored alive in vials or small plastic containers. To maintain a humid and habitable environment for the live specimens, the containers were filled with moistened pieces of decaying wood, fungus and moss. Specimens were retained in their original containers and kept alive in the laboratory at room temperature (22–23°C) in dark cabinets for observation and experimentation. Moisture levels were monitored and, if the substrate in the containers was almost dry to the touch, deionised water was added to maintain a mesic environment.

We recorded natural history observations in the field during Spring 2017 and recorded the arrangement of individuals in pinwheels with in situ photographs and illustrations. Millipedes were observed for a minimum of 10 minutes and observations were made by documenting the arrangements, behaviour and interactions amongst individuals during this period. Notes and drawings were recorded on collection cards printed on acid-free 100% cotton paper and included the following information: a unique collection code, state, county, locality description, global positioning system (GPS WGS84) latitude, GPS longitude, barometric elevation or GPS elevation, number of GPS satellites, GPS accuracy, collecting method, date, time, habitat and collectors. The collection card also included the sex, abundance (if more than one individual) and developmental stage of specimens and (if present) pinwheel diameter, number of individuals within the pinwheel and the sex and developmental stage of individuals composing a pinwheel. The sex, life stage and ring count of individuals were recorded in the field and then confirmed in the laboratory (Table 2). We used a chi-squared "goodness of fit" test using the totals of males and females in the pinwheels to test a significant difference from the null hypothesis of a 1:1 sex ratio. For this study, the term “ring” is used to refer to diplosegments. Counts of the body rings include the collum, haplosegments 2 - 4 and the telson. Moulting was observed in 10 individuals. These individuals were selected because of a characteristic pre-moulting appearance, where their bodies appeared swollen, particularly in the posterior rings. These moulting individuals were observed for 3–5 days. Each specimen was assigned a globally unique identification (GUID) catalogue number. Individuals from pinwheel aggregations were given a GUID beginning with "BLCV-" followed by a four-digit identifier for the pinwheel, then a three-digit identifier for the individual. (An exception, a sole individual collected from a colony in Arkansas was given an identifier beginning with "BLC" and followed by a unique two-digit identifier.) Solitary individuals (not collected from pinwheels) were given a GUID beginning with “MPE-” or “BLIV-” and followed by a unique four-digit identifier (Table 1). Material was fixed and preserved in 80% ethanol and deposited in the Virginia Tech Insect Collection (VTEC): https://collection.ento.vt.edu.

We used scanning electron microscopy (SEM) to examine adult morphology and document development from egg to adult. Specimens for imaging were fixed in 70% isopropyl alcohol, then air-dried at room temperature and humidity before mounting on 12.7 mm or 25.4 mm diameter aluminium SEM specimen mounts (“stubs”). We used 12 mm and 25 mm adhesive PELCO Tabs (Ted Pella, Inc.) or graphite conductive adhesive #112 (Electron Microscopy Sciences) to attach specimens to the stubs. Specimens were sputter-coated with a 20-nm thick layer of a mixture of platinum and palladium metals in a Leica EM ACE600 High Vacuum Coater. Images were acquired using a FEI Quanta 600 FEG environmental scanning electron microscope at the Virginia Tech Institute for Critical Technology and Applied Science.

The cuticle of *B. lecontii* is transparent, thereby allowing examination of internal anatomy such as defence glands with light microscopy and photography and without the need to enzymatically clear tissues. To photograph live specimens, a Canon 6D digital camera with 65 mm and 50 mm lenses attached to a Visionary Digital Passport II system (Dunn Inc., Charlottesville, VA) was used. A Leica M125 stereomicroscope and Zeiss Axio Imager 2 light microscope was used to examine defence glands, internal organs and gut contents of dissected specimens. Images were taken on the Zeiss microscope with a Zeiss Axiocam ERc 5s camera and Axiovision imaging software (AxioVs40 V 4.8.2.0 Carl Zeiss MicroImaging, Germany). Specimens were killed by freezing for 5–10 minutes at -20°C before examination. To observe gut contents, specimens that had been observed feeding on fungi were frozen, then fixed in alcohol. Adobe Illustrator CS6 and Adobe Photoshop CS6 were used for illustrating morphological features.

To quantify the colour of *B. lecontii*, reflectance spectra were measured from 18 live specimens (eleven females, seven males) from seven localities in Arkansas, Missouri, Tennessee and Virginia (all DAH-2017: 0512-02, 0513-02, 0516-01, 0516-02, 0517-01, 0517-02, 0521-02). A spectrometer, illuminated with a deuterium-halogen light source through a 400-μm diameter core fibre reflectance probe with a 24.8° acceptance angle, was used to measure percent reflectance or empirical measurements of reflectance intensity (300–700 nm) that are normalised by the intensity of a white standard (Ocean Optics USB4000 spectrometer, Ocean Optics QR400-7-UV reflectance probe, Ocean Optics WS-1 white reflectance standard). Specimens were held 6 mm from the end of the spectrometer probe and orientated so that the widest section of the dorsal surface of specimens were at normal incidence to the spectrometer probe to ensure standardised colour readings. The relationship between the percent reflectance by wavelength was graphed using the R (version 3.2.2) package pavo (version 1) (Maia et al. 2013, R Core Team 2016).

## Results

### Life History

**Figure 3.**
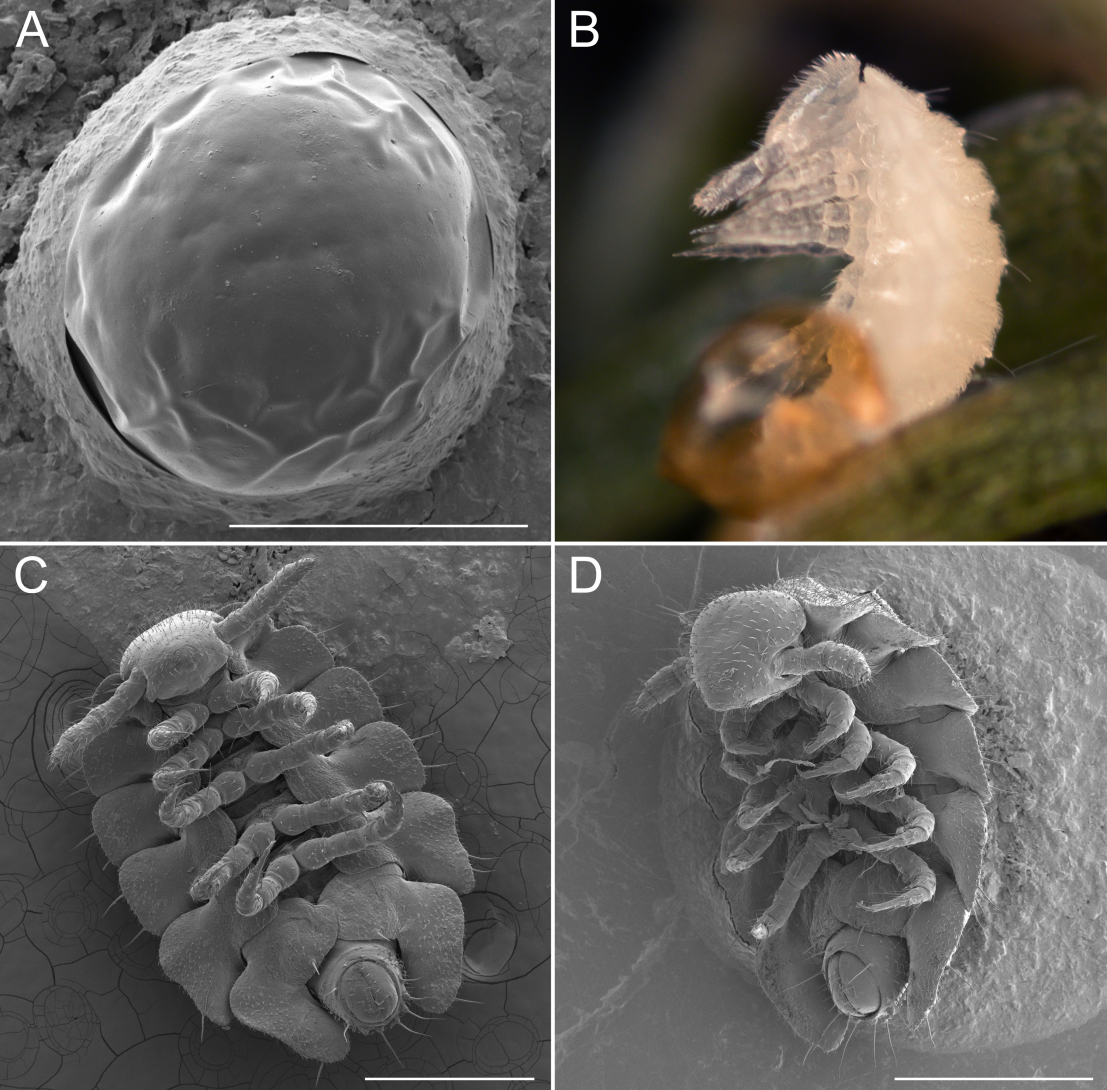
Early developmental stages of *Brachycybe lecontii.* **A.** Egg of *B. lecontii*, aside from some wrinkles due to desiccation, the egg surface is smooth (note: base of egg circled with ring of carbon paste); **B.** A hatching *B. lecontii* with five leg pairs and seven body rings, the amber oval object in the foreground is the empty egg shell; **C.** Stadium I *B. lecontii*, ventral; **D.** The same, left ventrolateral view. Scale bar A = 0.4 mm; scale bar C, D = 0.5 mm.

In the study of millipedes, an instar is referred to as a stadium (pl. stadia); for example, there are juvenile stadia (numbered I–VIII) that ultimately develop into an adult. On 13 and 16 May 2017, stadium I juveniles and males brooding eggs were observed in the field at two localities (Tennessee and Arkansas collection codes: DAH-2017-0513-02 and DAH-2017-0516-01). Eggs were 6–7 mm in diameter, light orange in colour and with no apparent surface sculpturing (Fig. 3A). The eggs from both of these localities hatched on 24 May, 11 and 8 days after they were observed in the field, respectively. A colony of live adult *B. lecontii* from the Arkansas locality (DAH-2017-0516-02) was maintained in the lab and two separate clutches of eggs were laid on 24 May and 2 June. The 24 May clutch hatched on 16 June (24 days after laying) and the 2 June clutch hatched on 27 June (26 days after laying).

**Figure 4.**
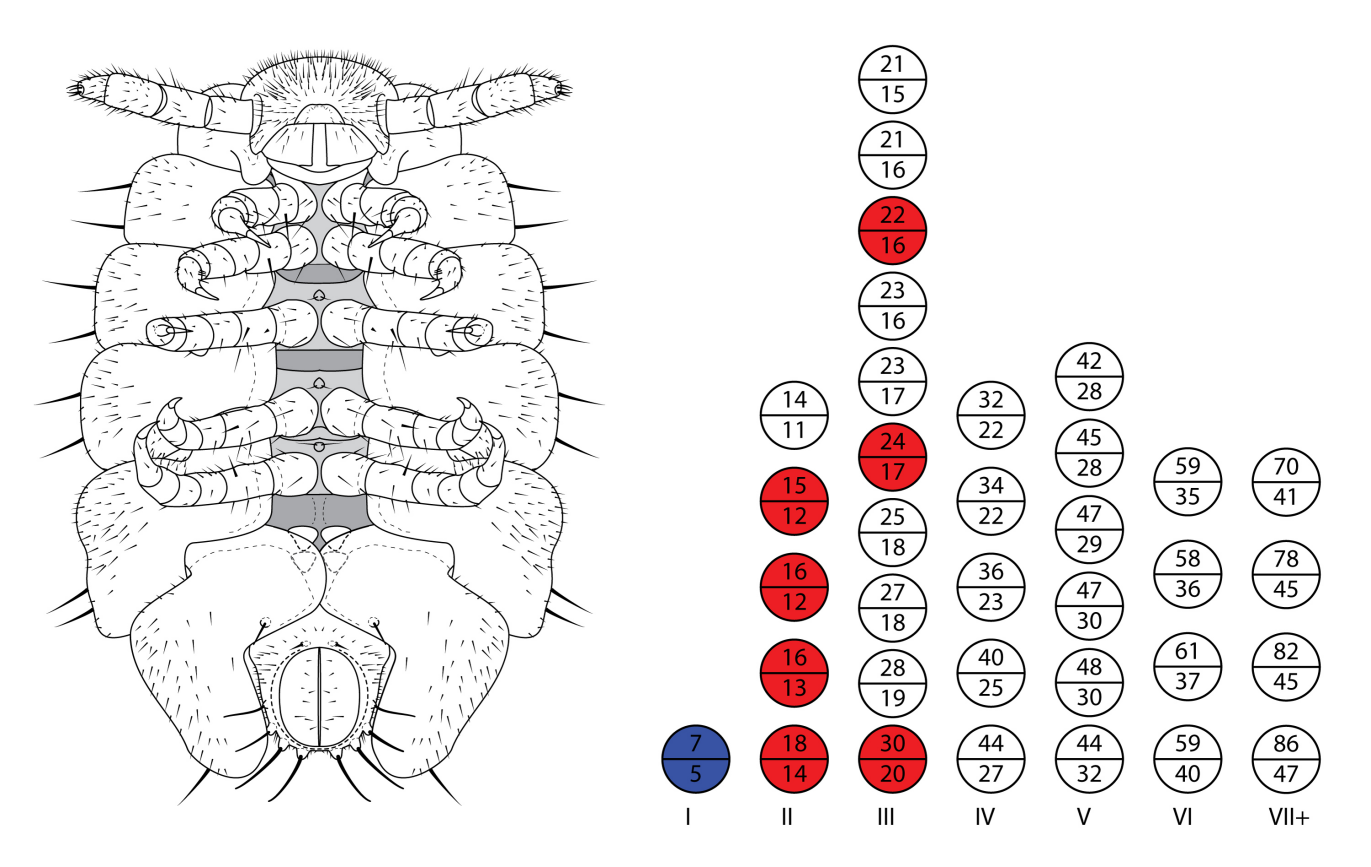
Development of *Brachycybe lecontii*. (Left) Illustration of a stadium I *B. lecontii*. (Right) Numbers of rings and legs observed in each developmental stage (stadia I–VII), VII+ denotes either stadia VII or VIII; top half of circle = number of rings; bottom half of circle = leg number (white, n = 1; red, n = 2–5; blue, n = 50).

**Figure 5.**
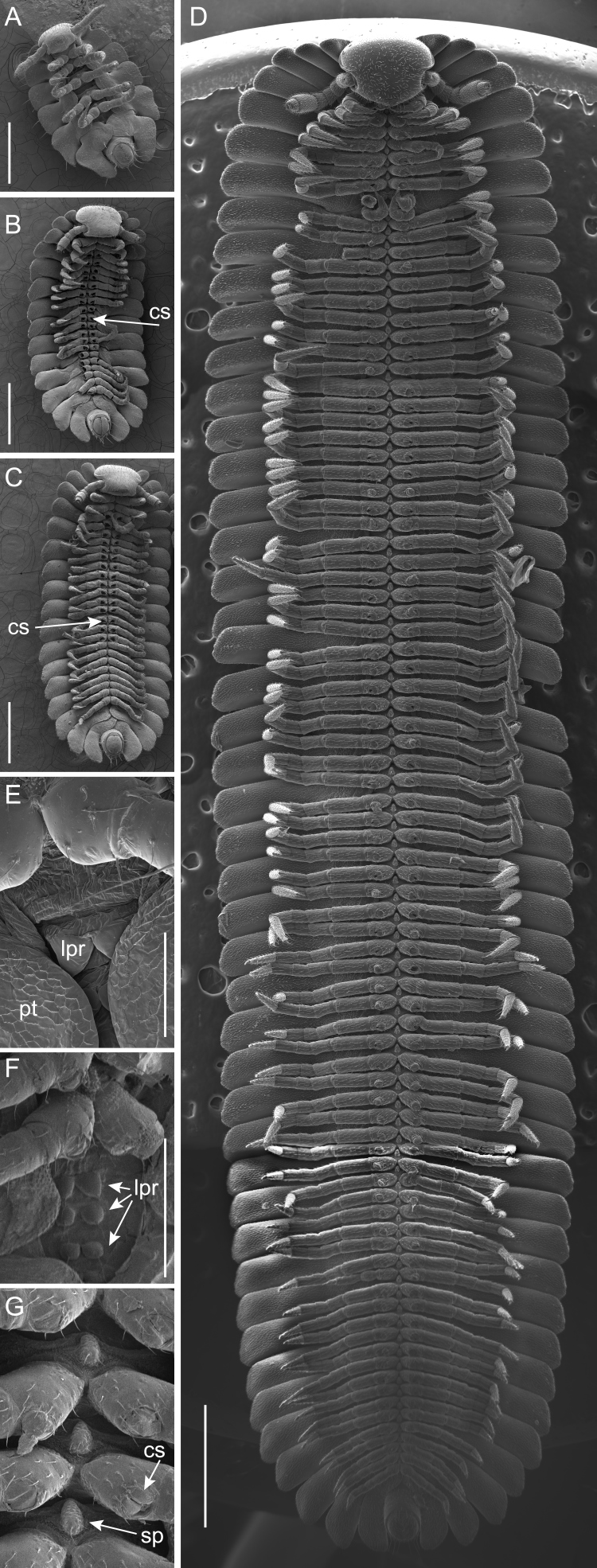
Scanning electron micrographs of stadium I, II and adult *Brachycybe lecontii* individuals, ventral view. **A.** Stadium I individual; **B.** Stadium II individual with coxal sacs (cs) visible; **C.** Stadium II individual; **D.** Adult male individual; **E.** Stadium I individual with two-leg pair rudiments (lpr) visible between pleurotergites (pt); **F.** Adult with three-leg pair rudiments visible between pleurotergites; **G.** Adult with coxal sacs on legs showing bivalve covering, left coxal sac expanded. Median sternal process (sp) visible between leg coxae. Scale bar A–C = 0.5 mm; scale bar D = 1.0 mm; scale bar E = 0.1 mm; scale bar F, G = 0.25 mm. Abbreviations: cs, coxal sacs; lpr, leg-pair rudiments; pt, pleurotergites; sp, sternal process.

Eggshells were not consumed by the newly-hatched stadium I individuals, nor were they consumed by any mature *B. lecontii* individuals. Of 50 stadia I individuals examined, all were 1.5–2 mm in length and had seven rings and five pairs of walking legs (Fig. 3B–D). Thereafter, the number of rings and leg pairs varied amongst individuals of the same life stage from stadium II onwards to the adult stage (Figs 4, 5A–F). The transition of leg pairs 9 and 10 from walking legs to gonopods in males could be discerned at a minimum of 24 rings (observed in individuals BLCV0005-006). The gonopods were observed to be fully sexually developed at a minimum of 35 rings (BLCV0004-025) (Fig. 1C, Fig. 5D). Dissection of females to determine the presence of mature eggs in the ovaries indicated that sexual maturity occurred at a minimum of 39 rings (BLCV0005-001).

Moulting was observed in 10 individuals. The individuals moved either to the bottom of their containers or into a crevice in the substrate where they curled up for a period of 3–5 days. The process of moulting lasted 1–2 days and was only observed in eight individuals since two were hidden in the substrate and were concealed from view. The dorsal side of the exuvium splits longitudinally, roughly halfway down each individual’s body; maintaining a curled position, individuals then wriggled out from the exuvium. Freshly moulted individuals were soft and fragile but, when prodded with a paintbrush, were capable of locomotion within a day of emerging. New rings were visibly whiter in colour and the defensive glands contained no visible secretions. Cuticular colour darkened and gland contents were present within two weeks. We did not observe *B. lecontii* constructing moulting chambers or consuming shed exuvia.

*Brachycybe lecontii* has coxal sacs that first develop in stadium II on leg pairs 3–5 (Fig. 5B). The coxal sacs are concealed by a clamshell-like pair of valves and open when the sacs are everted (Fig. 5G). During development and after moulting, coxal sacs were frequently not visible on newly-added leg pairs (Fig. 5C). Of 24 specimens examined, 23 had some legs without coxal sacs (always the posteriormost legs), with the exception of one adult male that had coxal sacs on all walking legs (except leg pair 1 that in Colobognatha is always lacking coxal sacs). In one specimen, asymmetric development of coxal sacs was observed and the left leg developed a coxal sac prior to the right one.

### Paternal Care

**Figure 6.**
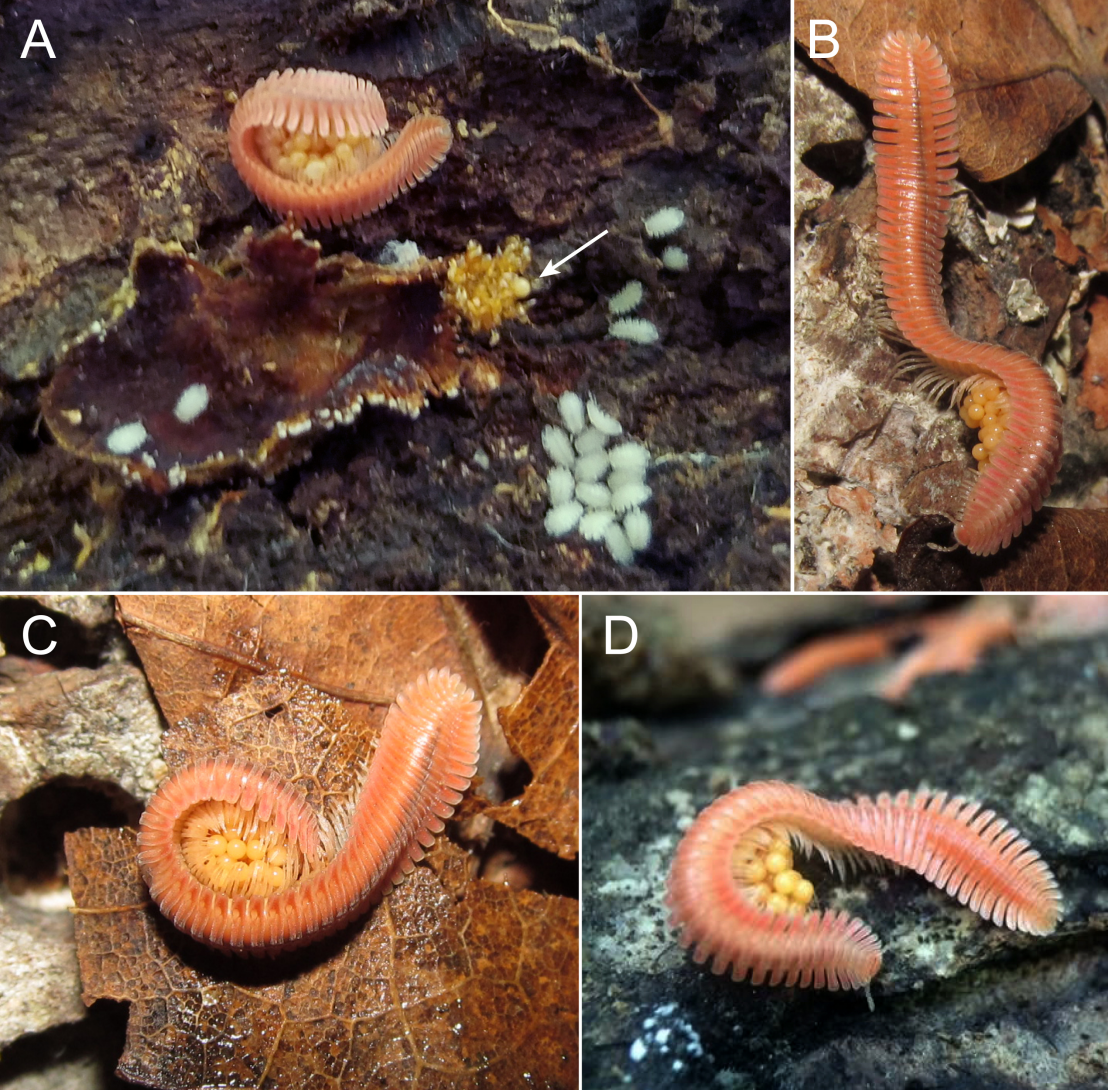
Paternal care in *Brachycybe lecontii*, males with clutches of eggs. **A.** Male curled around eggs with an aggregation of hatchlings near their natal site (arrow, one unhatched egg next to empty egg shells), from Gillam Park, Little Rock, Pulaski County, Arkansas; **B.** Male carrying eggs; **C.** Male curled around eggs. (B and C from Mount Kessler, Fayetteville, Washington County, Arkansas); **D.** Male curled around eggs from Campbell County, Tennessee (photo by Matt Berger).

Male *Brachycybe lecontii* with eggs were observed in the field in Tennessee and Arkansas at the localities DAH-2017-0513-02 (BLIV0010) and DAH-2017-0516-01 (BLIV0037). Males were observed caring for eggs in close proximity to the main colony. Subsequently in the lab, three additional males from an Arkansas locality possessed clutches of eggs (individuals BLIV0050-0052 from DAH-2017-0516-02). The males curled around the clutches of eggs with most of their body length, but with some posterior legs anchoring the brooding male to the substrate (Fig. 6). When lightly prodded with a paintbrush, the males would curl tighter around their eggs, but would not abandon the clutch. When exposed to bright light from the fibre optic light source of a microscope, the males would crawl towards the shade, with the anterior portion of the body being used to walk and the posterior half remaining wrapped around the eggs (Fig. 6B).

When artificially separated from their eggs (ca. < 2 cm), the males would seek them out and collect them. Males did not move far from the original location of brooding when seeking out the eggs and would frequently fail to find and collect eggs, if relocated further than 2 cm away from the millipede. Non-brooded eggs consistently did not hatch. Most brooded eggs successfully hatched, with 0–2 non-viable eggs observed per clutch (Fig. 6A). In the field, no adults were seen brooding juvenile millipedes. In the lab, adult males with clutches were observed to stop brooding and move away from the natal site once eggs in the clutch began to hatch.

Males did not appear to discriminate between eggs from their own clutch and eggs from other males’ clutches. In a laboratory colony, one male (BLIV0051) abandoned his clutch; subsequently, a second male—also with a clutch (BLIV0052)—collected BLIV0051’s eggs, thereby increasing his own clutch size to 115 eggs. Observed clutch sizes, excluding the aforementioned combined clutch, ranged from 54 to 70 eggs and average clutch size (including the aforementioned combined clutch) was 59 eggs (n = 5).

### Defence

**Figure 7.**
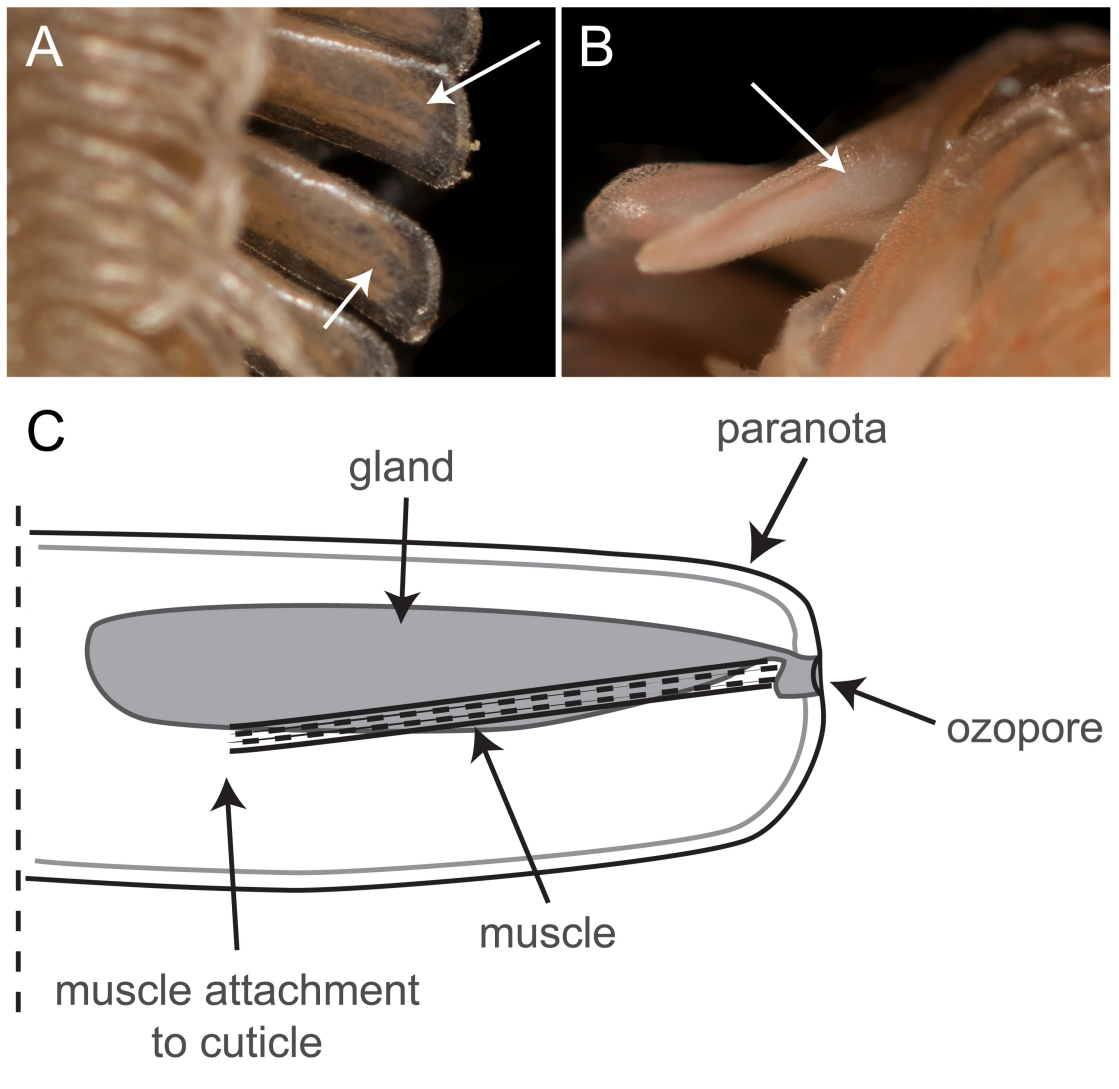
Chemical defence glands of *Brachycybe leconti*. **A.** Ventral view of the paranotum of a live *B. lecontii* (BLIV0080) that shows the vase-shaped defence gland (arrows) with bubbles of defensive secretion visible within the gland; **B.** Anterior view of live *B. lecontii* (BLIV0080), head bent downwards, which shows the large vase-shaped defence gland that is visibly white in colour and indicated by an arrow; **C.** Illustration (dorsal view) of the right paranotum of a *B. lecontii* individual.

Like other platydesmid millipedes, *B. lecontii* individuals have a type 2 defensive gland architecture (Eisner et al. 1978), which consists of a single chamber containing the liquid defensive secretions and a duct leading from the chamber to an ozopore, the opening of the gland (Fig. 7). We found the defensive glands to be large, occupying up to a third of the paranotal volume (Fig. 7B). The defensive chemical is liquid and has a bubbled emulsion-like appearance when the glands are viewed from outside of the cuticle (Fig. 7A). When millipedes were disturbed, the defensive chemical was secreted from the ozopores in discrete droplets. After oozing from the ozopore, the droplets from adjoining paranota coalesced to form a single clear, colourless droplet. The millipedes appeared to be able to control the location where defence secretions emanated and individual ozopores were triggered, based on the location of disturbance along the length of the trunk and from the left to right sides.

Stadia I *B. lecontii* possessed visible ozopores (Fig. 3D), but defensive compounds were apparently not present (no secretions visible within the glands or emitted as defensive secretion). In stadia II millipedes, defensive compounds were visible in the paranota, but disturbance of individuals with forceps failed to produce visible secretions (Fig. 5B, C). Obvious secretions (both visible within the glands and externally) were observed from stadia III onwards.

When individuals in pinwheels were disturbed with a paintbrush, defensive secretions were not readily produced. Further probing with a paintbrush caused the pinwheel members to recoil, break formation and disperse, but only occasionally would they produce defensive compounds. While disturbing some individuals in pinwheels, we did not observe any reaction in other non-disturbed members of the same pinwheel.

In 2016, during field observations, we observed that, when the adults were removed from a pinwheel containing both adults and early-stadia juveniles, an ant (*Camponotus* sp.) grabbed a juvenile in its mandibles and ran off, carrying the juvenile millipede with it. It is unknown whether the juvenile millipede was subsequently killed and consumed. In 2017, no predation on *B. lecontii* or interactions between *B. lecontii* and other animals were observed. In Arkansas (DAH-2017-0516-01), we observed a *B. lecontii* individual (BLIV0027) walking in the leaf litter and encountering several ants. The ants neither attacked nor investigated the millipede, even though it came into contact with the ants several times. At different sites in Tennessee and Arkansas (TN: DAH-2017-0512-02, DAH-2017-0513-02; AR: DAH-2017-0516-01), pinwheel aggregations and individuals were found on logs also inhabited by termites.

### Feeding

**Figure 8.**
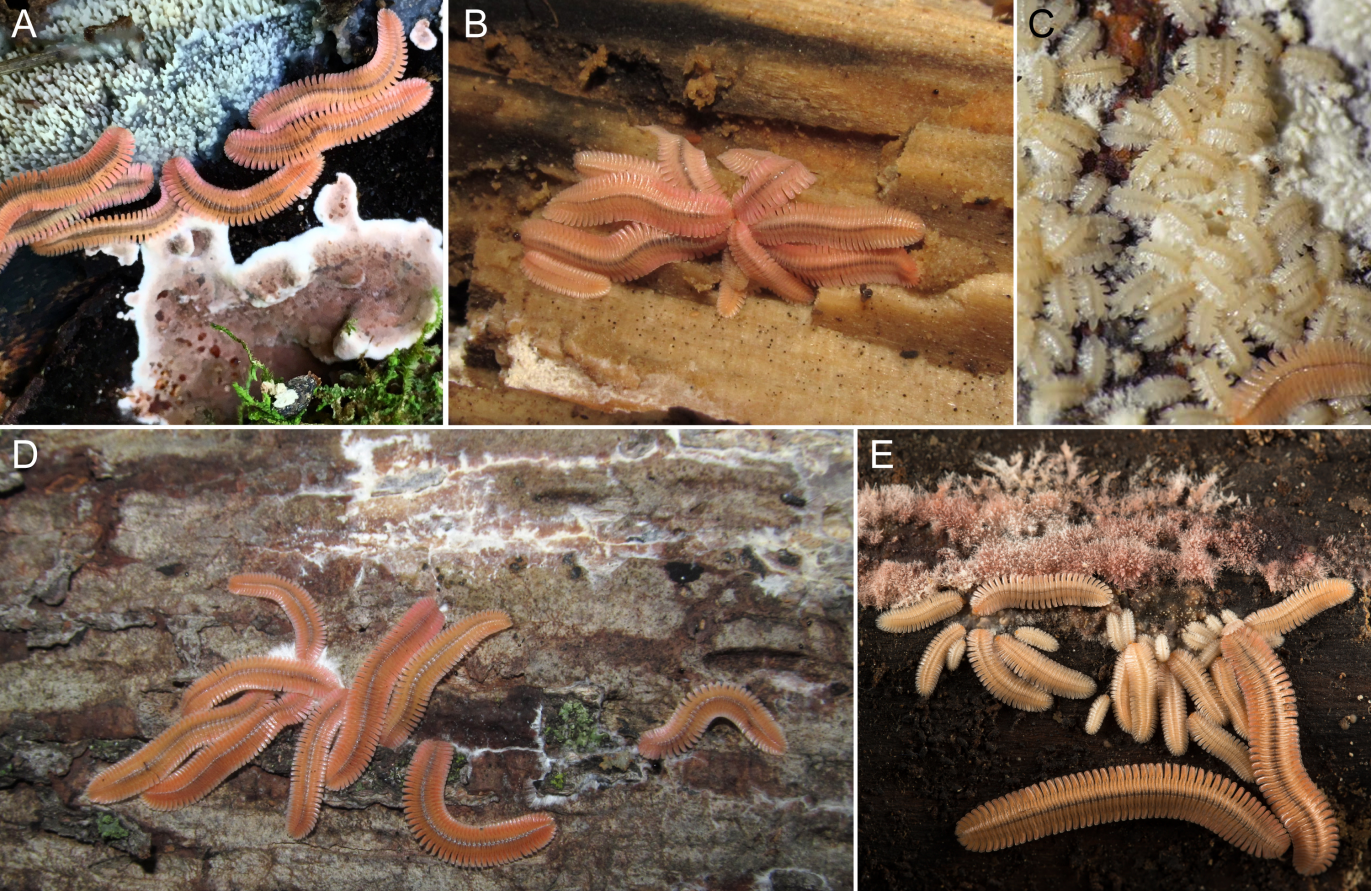
Pinwheel aggregations of *Brachycybe lecontii* millipedes with fungus. **A.** Millipedes with unidentified phlebioid polypore (top of frame) from Arkansas; **B.** Pinwheel of millipedes from Dickson County, Tennessee; **C.** Stadium I millipedes with unidentified fungus from Campbell County, Tennessee (photo by Matt Berger); **D.** Millipedes with unidentified fungus from Mount Kessler, Fayetteville, Washington County, Arkansas; **E.** Millipedes with unidentified fungus from Issaqueena Falls, Oconee County, South Carolina.

**Figure 9.**
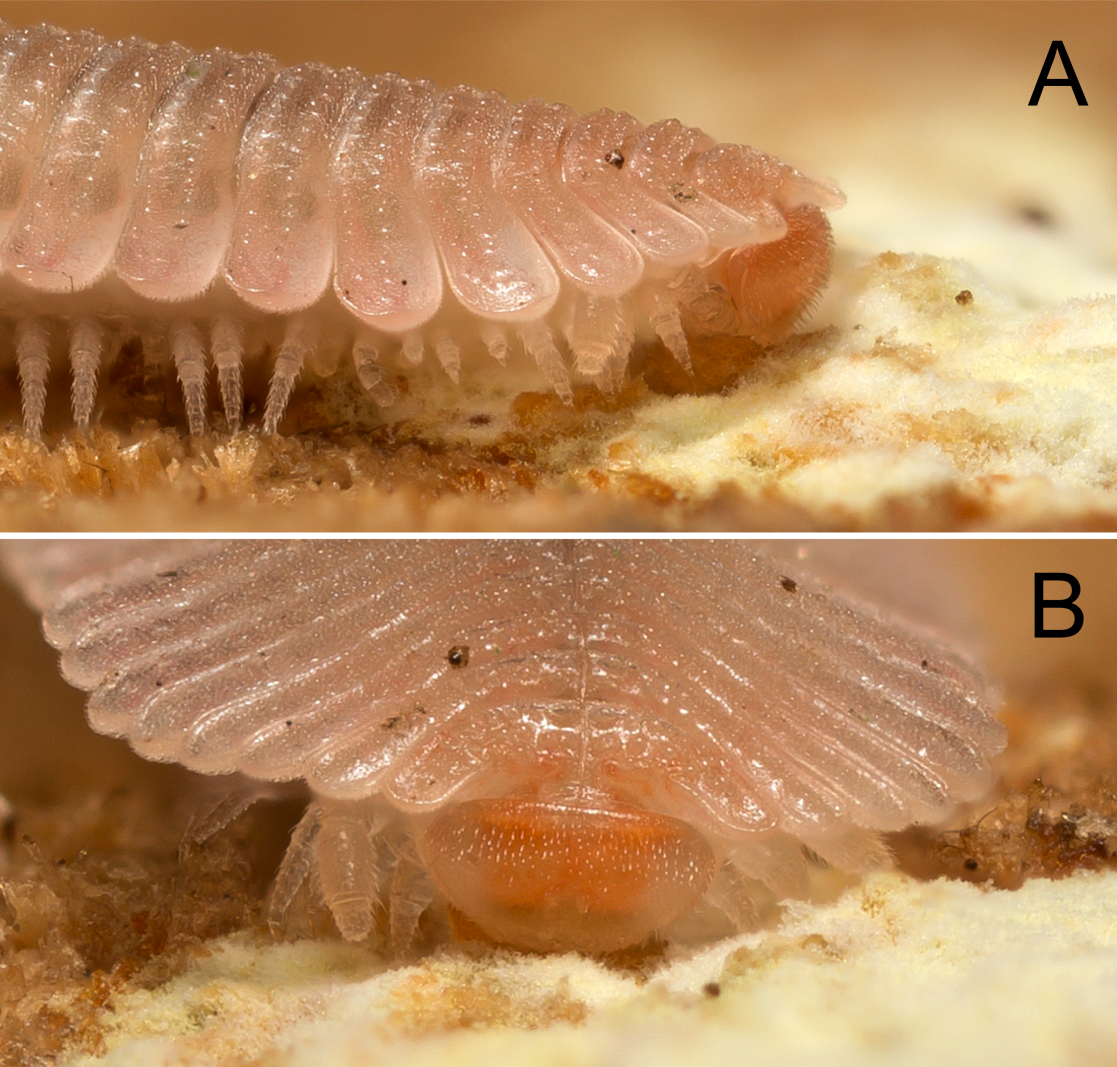
*Brachycybe lecontii* with fungus. **A.** Lateral view of head and first 10 rings of *B. lecontii* with fungus; **B.** Anterior view of the head and first 8 rings of same individual as above with fungus.

**Figure 10.**
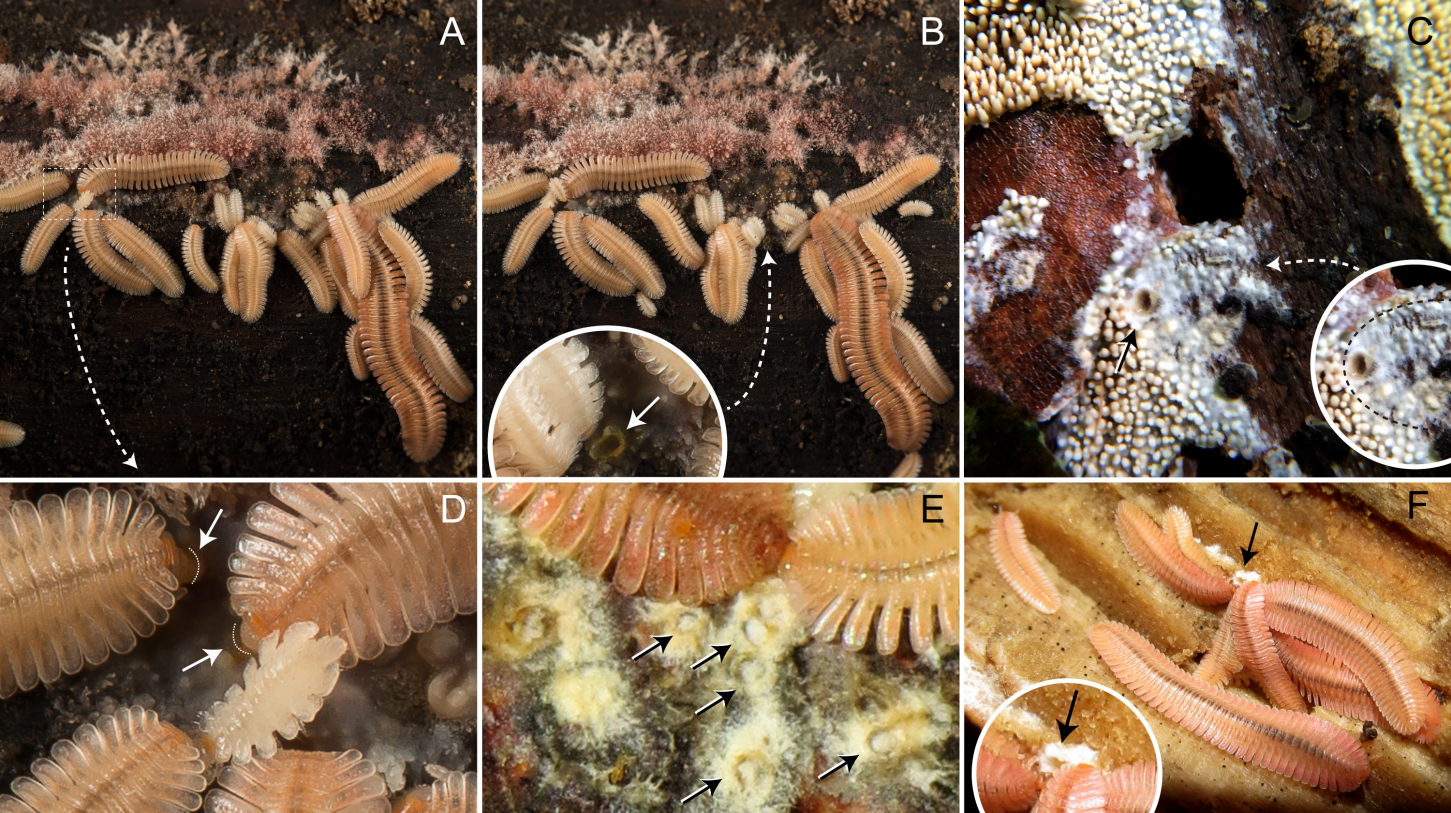
Evidence of fungal feeding in *Brachycybe lecontii*. **A.** Millipedes with unidentified fungus from Issaqueena Falls, Oconee County, South Carolina; **B.** Same after 15 minutes with evidence of fungal feeding from a pit (feeding bowl) in the surface of the fungus (inset, magnified view); **C.** Feeding bowl, arrow and impression of the anterior section of the millipede trunk (impression outlined in black dotted line, inset); **D.** Magnified view of A, showing two millipedes with their heads immersed in gelatinous fungal tissue, arrows and curved dotted lines showing fungus that is bulging out of the feeding bowl; **E.** Several feeding bowls in fungus, arrows; **F.** Millipedes with feeding bowl, arrow, in fungus from Montgomery Bell State Park, Dickson County, Tennessee (feeding bowl impression, inset).

*Brachycybe lecontii* were often found associated with fungi. During fieldwork in 2016, we collected *B. lecontii* with fungi in the order Polyporales, genera *Irpex* and *Trametopsis*. In the field and laboratory, we observed *B. lecontii* with their heads held above or embedded in fungal tissue (Figs 8, 9). Pits or indentations in the surface of the fungus, henceforth called “feeding bowls”, were frequently observed when millipedes were removed from the fungal mats (Fig. 10). Feeding behaviour was discerned, based on the noticeable presence of fungus near the millipede and the position of the millipede's head in proximity to the fungal tissue. Feeding was observed at five of the seven locations in the field (Table 3).

We did not observe any visible trace of plant or fungal matter in the gut during dissections of ten specimens. We did not observe *B. lecontii* defecating solid faeces, though we twice observed *B. lecontii* defecating liquid. The liquid was an approximately 1 mm diameter droplet of colourless fluid. We also observed millipedes regurgitating clear liquid after submergence in 70% isopropyl alcohol for specimen preservation.

### Feeding Structures

**Figure 11.**
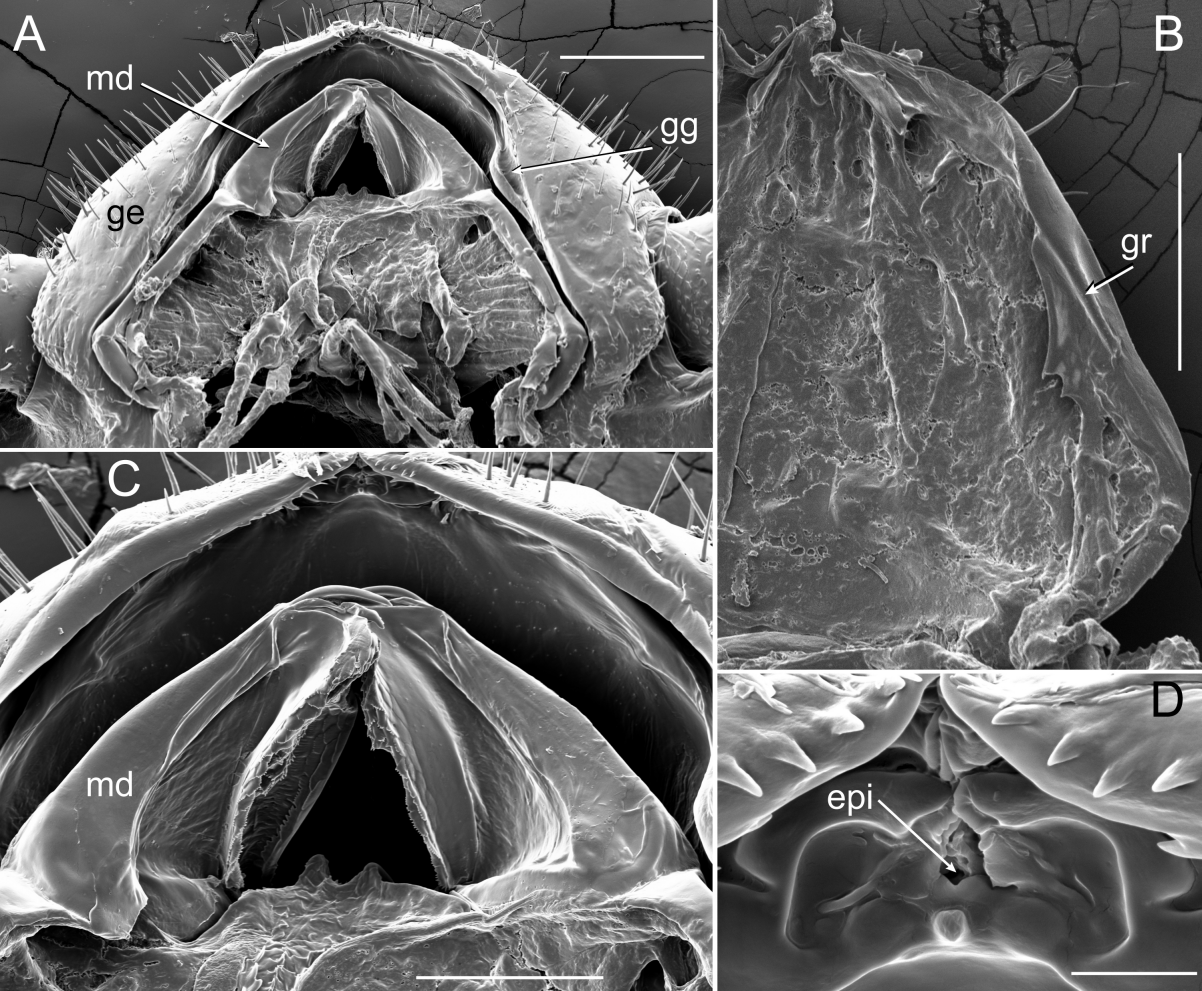
Mandibles and other mouthparts of *Brachycybe lecontii*. **A.** Ventral view of mandibles (md) and buccal cavity of *B. lecontii*, gnathochilarium removed; **B.** Gnathochilarium of *B. lecontii*, dorsal view of dissected gnathochilarium; **C.** Ventral view of mandibles, gnathochilarium removed; **D.** Ventral view of epipharyngeal cleft (epi), gnathochilarium removed. Scale bar A, B = 0.1 mm; scale bar C = 50 μm; scale bar D = 5 μm. Abbreviations: epi, epipharyngeal cleft; ge, genae of head; md, mandible gnathal lobe; gg, genal groove; gr, gnathochilarial ridge.

**Figure 12.**
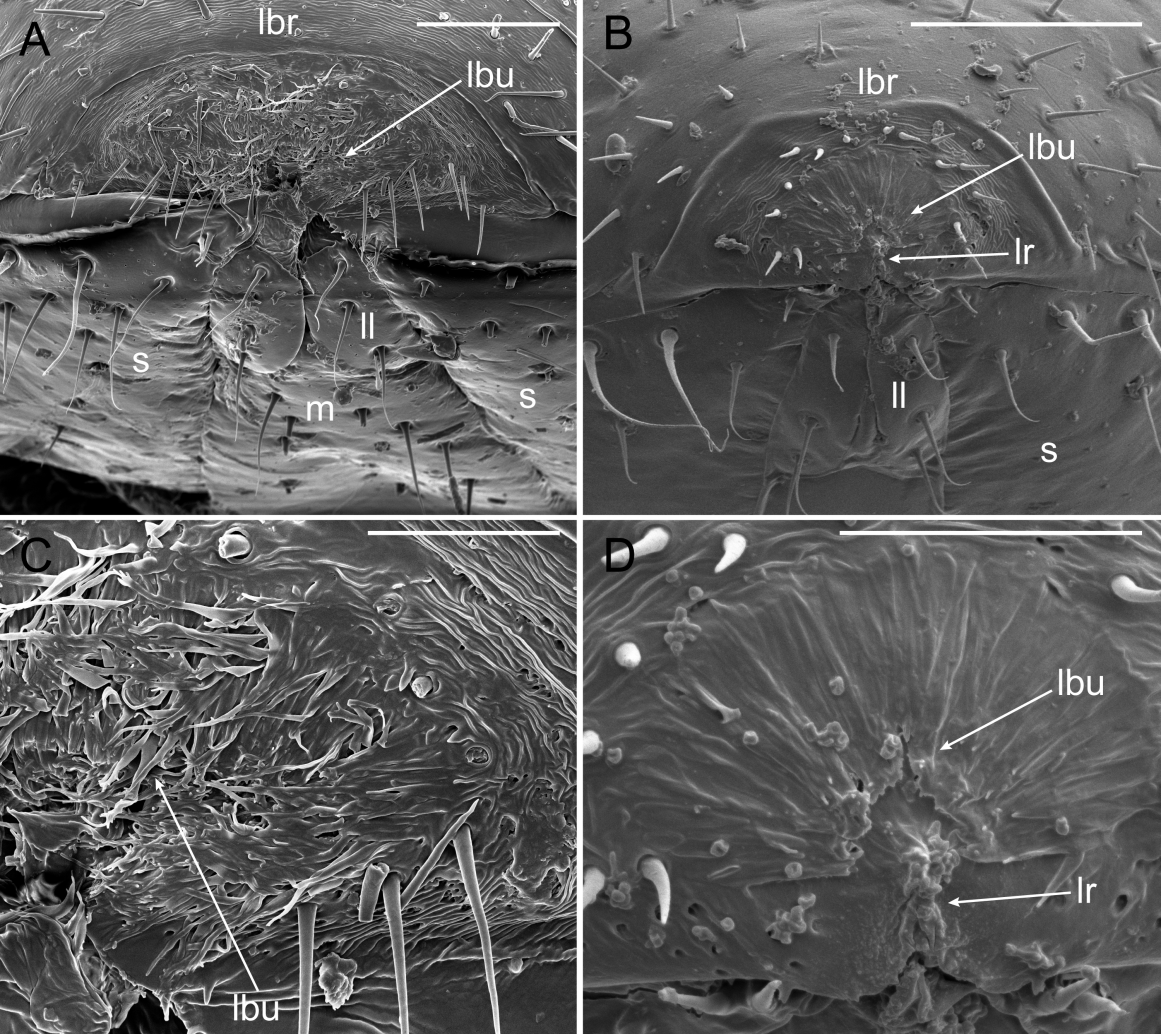
Labrum of *Brachycybe lecontii*. **A.** Fibrous brush-like labrum and gnathochilarium of an adult *B. lecontii*; **B.** The same of a juvenile *B. lecontii*; **C.** Magnified view of the labrum in A; **D.** Magnified view of the labrum in B. Scale bar A, B = 50 μm; scale bar C, D = 20 μm. Abbreviations: lbu, labral brush; lbr, labrum; ll, laminae linguales; lr, labral ridge; m, mentum; s, stipes.

The gnathochilarium and labrum of *B. lecontii* are tightly appressed with a thin crevice separating these structures. The mandibles, including their bases, were entirely entognathous and enclosed by the labrum, genae and gnathochilarium of the head (Fig. 11). The mandibles were simple with a flat occlusal surface and small external and internal teeth distally. They lacked a pectinate lamella and molar plate. The gnathochilarium possessed a ridge on the lateral margins that appears to fit into a corresponding groove on the head (Fig. 11A, B). The labrum was cleft in two and visible on the ventral side of the labrum as an epipharyngeal cleft (Fig. 11D). The exterior tip of the labrum was composed of a ramifying, fibrous cuticle (Fig. 12) with scattered sensilla and pits on the surface. This structure was roughly semicircular in shape with a radius of approximately 20 µm in a stadium I juvenile (Fig. 12B, D) and 50 µm in an adult male (Fig. 12A, C). The fibrous structure was concave and therefore not continuous with the overall convex head capsule. The outer semicircular rim of the fibrous structure was wrinkled in appearance with fibrous strands emanating ventrally towards the gnathochilarium. These brush-like fibrous strands pointed inwards towards the centre of the labrum (Fig. 12C). In the stadium I individual, the fibrous strands at the centre of the labrum were raised into a ridge, about 10 µm in length and vertically orientated (Fig. 12D). In several older individuals, this fibrous labral structure and surrounding setae appeared frayed and worn, with setae on the structure broken or missing. In stadium I individuals, the structure showed less wear.

### Pinwheeling

The formation of pinwheels was associated with the presence of fungus. In the laboratory and on occasions when no fungus was present, millipedes would aggregate, but would not assemble into pinwheels. The centres of pinwheels were frequently atop the fungus. Of the nine pinwheels observed in the field, six of them (BLCV0001–3, BLCV0007–9) included millipedes with their heads embedded in fungus (Suppl. material 1). Three pinwheels (BLCV0004–6) had fungus present elsewhere on the branch or limb upon which the millipedes were aggregated.

Pinwheels persisted for several weeks. In the laboratory, a *B. lecontii* colony collected in the field from an Arkansas site (MK-2016-010) maintained a pinwheel for 27 days, from 23 May to 18 June. Individuals entered and exited the pinwheel, but the overall formation, particularly the location of the central hub, remained in a constant location.

**Figure 13.**
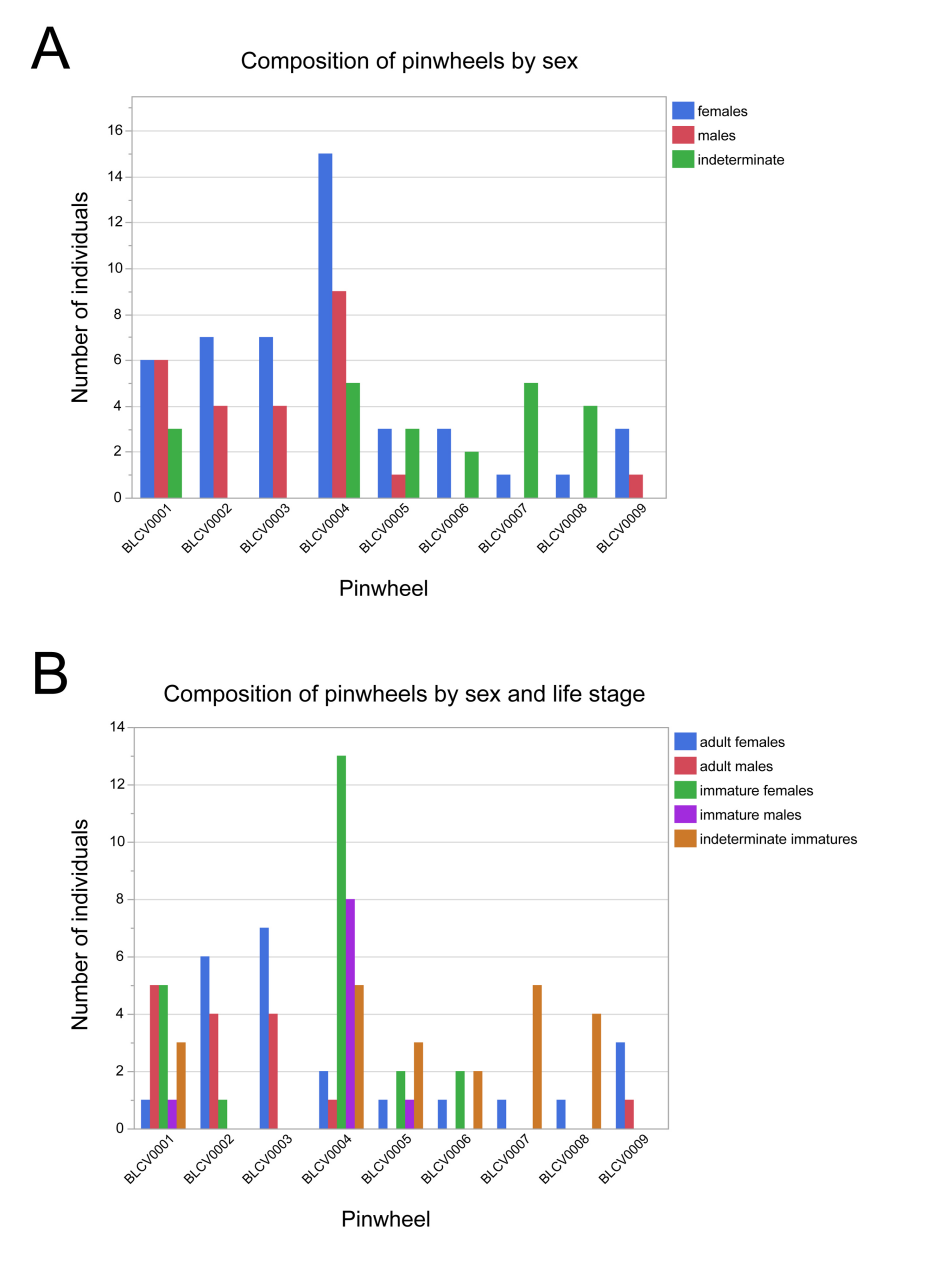
Composition of social pinwheel aggregations of *Brachycybe lecontii*, according to sex and life stage. **A.** Composition of pinwheels by sex; **B.** Composition of pinwheels by sex and life stage. BLCV numbers correspond to individual pinwheels.

Individuals in pinwheels varied in sex and developmental stage (Fig. 13A). Although pinwheels often consisted of numerous juveniles that could not be sexed, amongst those individuals whose sex could be determined, the pinwheels had a significant difference in sex proportions with consistently more females than males (Fig. 13B). A chi-squared test of "goodness of fit", using the totals of males and females in all nine observed pinwheels, found a significant difference from the null hypothesis of a 1:1 sex ratio (X^2^ = 6.21, df = 1, p = 0.013).

At one locality (DAH-2017-0516-01), over a dozen pinwheels of stadia I and stadia II *B. lecontii* were observed. These were not collected as sex could not be determined from early-stadia individuals. Stadia I and stadia II juveniles were also observed pinwheeling with mature females of *B. lecontii* on fungus and when the adults were collected, the juveniles dispersed (locality BLIV0039-0040, AR).

### Leg Morphology

**Figure 14.**
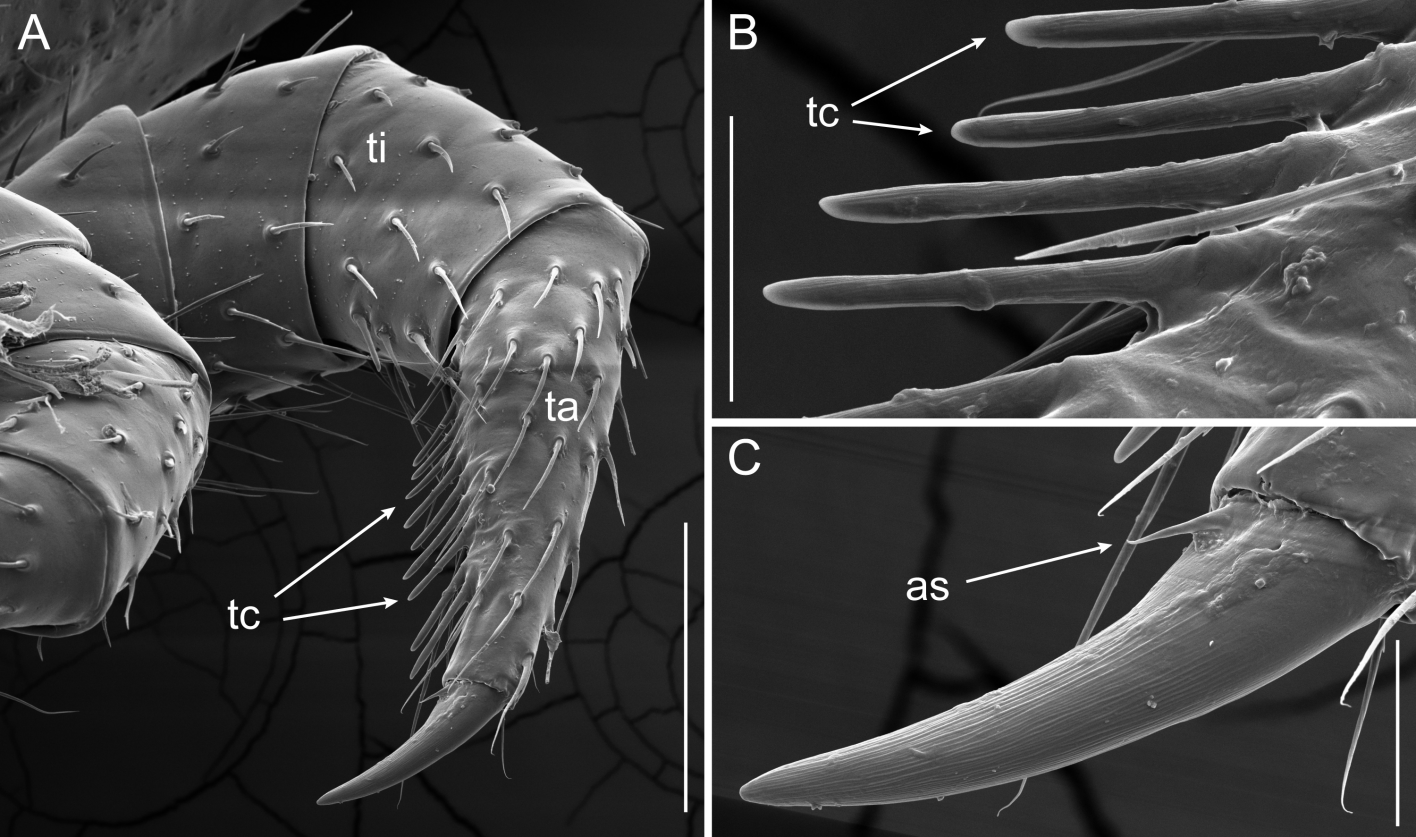
Scanning electron micrographs of combs present on the tarsus of *Brachycybe lecontii* (anterior view). **A.** Tarsus of adult *B. lecontii* with comb-like structures; **B.** Magnified view of tarsal comb; **C.** Magnified view of tarsal claw. Scale bar A = 0.1 mm; scale bar B, C = 20 μm. Abbreviations: as, accessory seta; ta, tarsus; tc, tarsal comb; ti, tibia.

**Figure 15.**
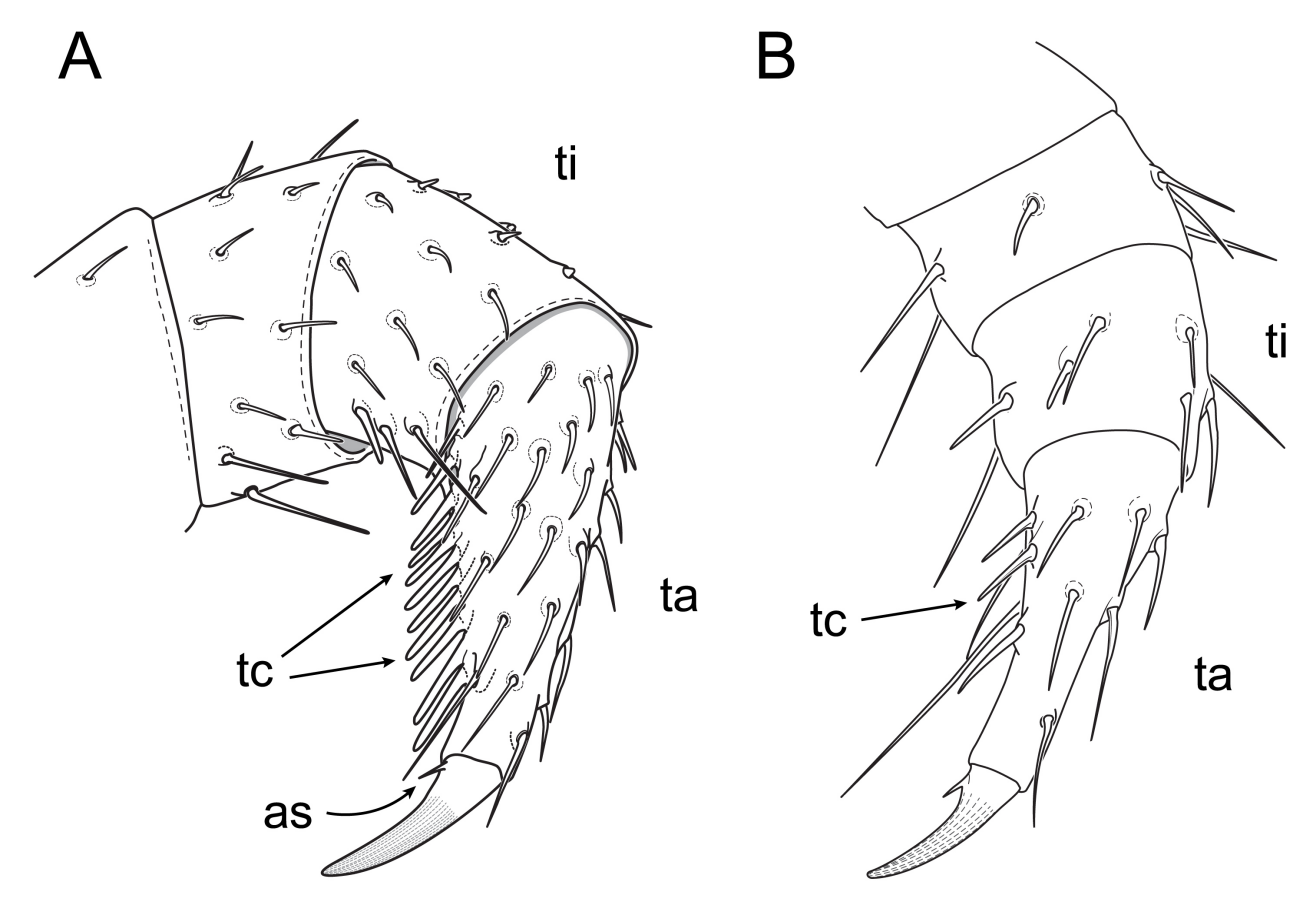
Illustrations of tarsal combs of *Brachycybe lecontii* (anterior view). **A.** Comb on second leg of an adult *B. lecontii*; **B.** Comb on the first leg of a stadium I *B. lecontii*. Abbreviations: as, accessory seta; ta, tarsus; tc, tarsal comb; ti, tibia.

A feature, consisting of a single row of comb-like setae on the tarsus, was found on the six anterior-most leg pairs of both male and female *B. lecontii*—referred to as a “tarsal comb” (Figs 14, 15). The tarsal claw of *B. lecontii* is armed with a small seta, situated at the base of the tarsal claw (Fig. 14C, Fig. 15A). On adults, the modified setae of the tarsal comb had short, thick shafts which flatten and terminate in blunt ends (Fig. 14B). Tarsal combs were visible as early as the first stadium. Although the undeveloped combs of juveniles lacked the flattened shaft and blunt tips present on the adults, they were recognised by their linear alignment in a single-row and stoutness, relative to other unmodified setae (Figs 14, 15). Larger, older millipedes possessed a greater number of setae consisting of the tarsal comb and thicker, longer setae than in younger millipedes (Table 4).

### Colour

**Figure 16.**
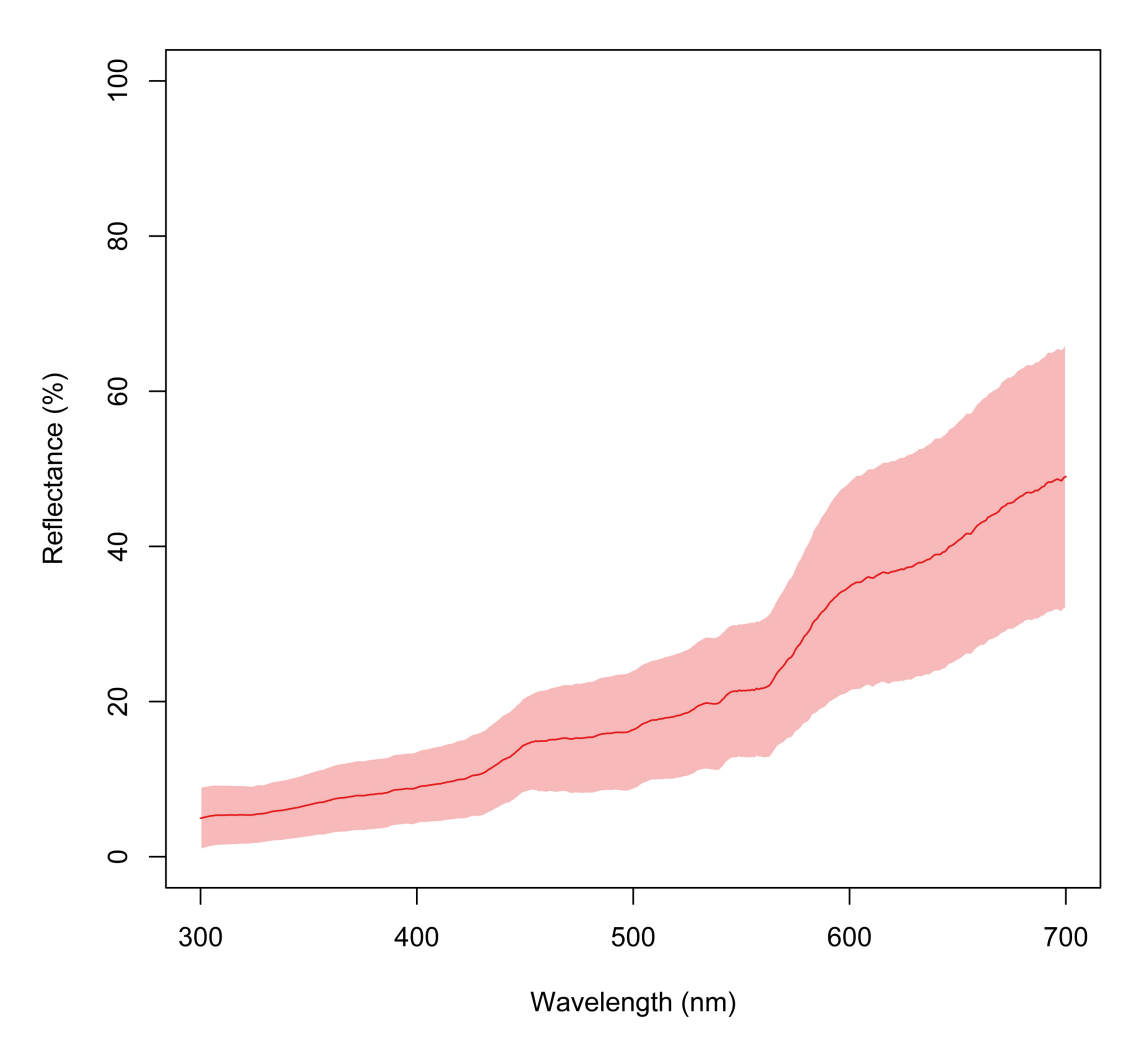
Reflectance spectra of the cuticle of *Brachycybe lecontii*. Reflectance (%) measured in 18 live millipedes. The y-axis is percent reflectance and x-axis is wavelength of light in nanometres (nm). The plot includes a median (red line) and standard deviation (light red area above and below the line).

Recently eclosed stadium I *B. lecontii* were translucent white in colour and, as the millipede aged, the colouration became pink (Fig. 3B, 8C). The mature millipedes were generally pink in colour with variations in hue from beige to deep salmon (Fig. 8). Body colouration was uniform within individuals, but posterior body rings are weakly pigmented after ecdysis and upon recent ring addition by anamorphosis. Preserved specimens in 70% isopropyl alcohol faded to a dull beige. Examination of reflectance spectra of live individuals revealed the least reflectance at short wavelengths, with a median of 4.97% reflectance at 300 nm (minimum 2.10%, maximum 19.53% Suppl. material 2). The highest percent reflectance occurred at longer wavelengths, with a median of 48.97% at 700 nm (minimum 15.59%, maximum 77.99%) (Fig. 16). Inflection points of the spectrum were present at 450 nm and 580 nm.

## Discussion

### Life History

Youngsteadt and McAllister (2014) found that *B. lecontii* millipedes eclosed from the egg after 21 days, but did not directly observe oviposition, potentially introducing some inaccuracy to conclusions about the incubation period. From our observations, we found that the oviposition and incubation periods of *B. lecontii* were similar to its sister species *B. nodulosa* from Japan. Murakami (1963) stated that the oviposition period of *B. nodulosa* extended from mid-May until late July, with an incubation period of 3–5 weeks at 13-24ºC. Clutches of *B. lecontii* eggs, laid in the laboratory, were observed hatching 24–26 days later. With our observations of an incubation period of 3–4 weeks at room temperature (20–25ºC) and because eggs and stadium I juveniles occur in the field as early as 13 May, the oviposition period for *B. lecontii* begins in mid-April and continues until early June. The eclosion from eggs in May by *B. lecontii* would synchronise the development of juvenile millipedes to coincide with warmer temperatures and potentially greater fungal growth. Winter behaviour and mating of *B. lecontii* were not observed, but are possibly similar to *B. nodulosa* which overwinter under logs and breed from May to July (Murakami 1962a). Murakami (1962a) found juvenile and adult stages of *B. nodulosa* overwintering (n = 1500) with the most individuals having 13, 20 and 41 rings. While there are a limited number of collections of *B. lecontii* in the winter that include habitat information, adult males have been found by the author, PEM in early February (2016) 10 cm below ground beneath a frozen humus layer.

Stadia were determined, based on the number of leg pairs lacking coxal (eversible) sacs. As coxal sacs are not present on newly-added rings immediately after ecdysis and the minimum number of rings added during moulting is constant, the stadium number can be determined by dividing the number of leg pairs without coxal sacs by those with and then rounding up. Stadium I juveniles possess five-leg pairs and seven body rings, which are not found in its sister species or any other millipedes, which typically have three- or four-leg pairs post eclosion (Murakami 1962b, Enghoff et al. 1993, Youngsteadt and McAllister 2014). Based on our observations, we found that stadium II individuals had 11–14 leg pairs and 14–18 rings; stadium III had 15–20 leg pairs and 21–30 rings; and stadium IV had 22–27 leg pairs and 32–44 rings (Figs 4, 5). Stadium IV male individuals were identified by the presence of gonopod primordia (the gonopods become distinguishable from walking legs at this stage) and the number of rings of stadium IV were determined from these individuals. This mode of development is similar to that found in *B. nodulosa*, where males are first discernible at stadium IV due to the presence of the gonopods (Murakami 1963). Assignment of stadium, based on leg pair and body ring counts, becomes challenging at older stadia, as the variation in the counts can widely overlap and the number of rings and leg pairs added do not appear to follow a consistent pattern and are not tightly correlated as they are in younger individuals. Moreover, an odd or even number of leg pairs may be present and an odd or even number of leg pairs may be added between moults, thereby hampering stadium calculation (Murakami 1962b, Enghoff et al. 1993). Variability in ring counts amongst stadia was lower in *B. lecontii* than in *B. nodulosa*, which may be due to the fewer number of specimens examined for *B. lecontii*. Approximately 200 *B. lecontii* were utilised for this study, with 10 specifically retained for observations on their moulting behaviour.

Frequently referred to as eversible glands, coxal sacs are found in many millipede taxa (Koch 2015) and may function to absorb water from their surroundings (Gill 1981). Coxal sacs were typically not present on newly-added walking legs. However, the presence of coxal sacs on all walking legs was observed in a single adult male *B. lecontii*. Because as colobognaths are euanamorphic (adding rings and legs for an indeterminate period of time, even past sexual maturity, Enghoff et al. 1993), the full complement of sacs is unlikely to be a feature of a final moult. Development of the coxal sacs takes longer than development of the legs between moults, hence the one-moult delay seen in *B. lecontii*. A delay in moulting for this individual, for reasons such as poor environmental conditions, may have allowed the development of the coxal sacs to catch up with the development of the legs. The apparent maturation lag of the coxal sacs, making the posterior-most leg pairs always lacking the structures, may be adaptive to avoid resorption of liquid faeces.

Moulting in *B. lecontii* occurs according to the same four stages observed by Murakami (1963) in *B. nodulosa*: prerigidation, rigidation, intermediate period and recovery. In *B. nodulosa*, prerigidation, a stage in which the body swells and the pre-anal ring protrudes, lasted up to a week; rigidation, where the millipede curls under the cover of substrate, also lasts up to a week; the intermediate period, when the actual process of ecdysis occurs, may last up to a day; and finally, the recovery period occurs over one to two weeks after the intermediate period. The maximum length of each stage in *B. nodulosa* is slightly longer than what we observed in *B. lecontii*, but may be dependent on environmental conditions and the vigour of the specimens.

### Paternal Care

Paternal care in *B. lecontii* was similar to that documented in *B. nodulosa* (Kudo et al. 2010). Brooding by males appears vital to the survival of eggs, since eggs that were separated from the brooding male were always unviable; however, we did not examine the cause of death for non-brooded eggs. A number of factors may lead to the death of eggs without paternal care. Fungal infection is likely, as Kudo (2011) found that eggs of *B. nodulosa* would become covered in fungal hyphae if not tended by a male. Kudo et al. 2010 suggested that males may prevent fungal infection by physically covering eggs with their body, actively removing fungi with their mouths or legs or secreting fungicides. Macias et al. (2019) found that the fungal community associated with *B. lecontii* was diverse and included a number of entomopathogenic species that would likely be harmful to eggs. Other factors of egg mortality, such as desiccation, are also likely to be involved. The failure of males to recover all of the eggs from their clutches, if they are separated from them, suggests an inability to evaluate clutch size or number of eggs. Alternatively, it may be more advantageous to not expend excess time and energy in collecting their whole dispersed clutch: lost eggs may have been preyed upon and thus not obtainable; and collection attempts could cause the male to wander away from safety and resources.

Unfortunately, the transfer of eggs from one male to another—whereby a clutch was increased in size to 115 eggs—was not directly observed in the process, so the mechanism is unknown. One male abandoned or may have accidentally lost his clutch and the eggs were collected by another male, despite our observations that males wrap tightly around their clutches and are not prone to uncurling. The male may have abandoned his clutch, though there were no apparent differences between eggs in the combined clutch. Abandonment of clutches by males was observed by Kudo et al. (2010) in *B. nodulosa* and by Gardner (1974) in *B. rosea* and *B. producta*, as a consequence of disturbance of the males by the researcher. Abandonment was not observed here in *B. lecontii* when males were disturbed by probing with a paintbrush. However, a different outcome may have occurred if a male were repeatedly or constantly handled or disturbed by other millipedes in the colony. Doubling the clutch number could also have been a theft of eggs from one male to be incorporated into the other clutch. Kudo et al. (2010) indicated that females (which notably lack eyes, as do all Platydesmida, Müller and Sombke 2015) are able to assess tergal width and therefore capability as an egg-brooder. Perhaps females are able to assess the ability of males to care for a large clutch of eggs. If this occurs, then multiple matings may occur, which is consistent with evidence of this type of reproductive strategy in other millipedes (Wojcieszek and Simmons 2012). Regardless of possible mechanisms or causes of brood transfer, the singular observation of clutch redistribution in a laboratory setting may simply be an anomaly, reflecting abnormal behaviour that would otherwise not be found in the wild.

### Defence

We found that the shape of the defence glands was different than that previously documented in literature. Although formerly described as "slender lengthy tubes" (Eisner et al. 1978: p. 48), we discovered that the glands were long and conical in shape and more voluminous than previously described, occupying from a quarter to a third of the paranotal volume (Fig. 7B). A possible explanation for this discrepancy in observations may be that previous authors examined live specimens that may have expended some or all of their defensive secretions or dead specimens stored in alcohol for a length of time, thereby causing the defensive glands to shrivel in the preservative.

Defensive secretions were absent in the defensive glands of stadium I individuals (though ozopores are present on the paranota) and do not appear to be produced in the gland until stadium II, during which they become visible within the gland reservoir. Stadium II individuals were not observed to discharge any chemical secretions despite disturbance with forceps, suggesting that stadia I and II millipedes may be unable to chemically defend themselves. By stadium III, however, millipedes were able to produce defensive compounds and discharge them through their ozopores in response to disturbance. The inability of early stadia millipedes to chemically defend themselves may necessitate aggregation behaviour with later stadia and adults that are chemically protected from potential predators.

Our field observations of the interactions between ants and *B. lecontii*, whereby chemically-defended adults of *B. lecontii* were avoided, suggests that their defensive chemicals, containing alkaloids (Shear 2015), may have similar anti-predator properties to buzonamine (Wood et al. 2000). Other arthropods are chemically defended by alkaloids such as ladybugs (coccinelline) and monarch butterflies (pyrrolizidine alkaloids); however, their predators are primarily avian (Kelley et al. 1987). Ants are the only observed predators of *B. lecontii* and their alkaloid is likely effective as a predator deterrent. A diversity of predators may exist and field studies of predation with cameras or clay models would help address likely receivers of the chemical.

*Brachycybe lecontii* has been found to produce an alkaloid (Shear 2015). Possible reasons, unrelated to defence for the production of the alkaloid, include production as an aggregation cue. For example, the defence chemicals of the Indian polydesmidan millipede *Streptogonopus phipsoni* contain benzaldehyde, which acts as an attractant to conspecifics (Bellairs et al. 1983). Additional chemical analyses from a wider range of localities and controlled field predation studies are required before any robust conclusions can be drawn regarding the function of *B. lecontii*’s alkaloid chemical defence.

### Feeding

Fungivory has been observed in several *Brachycybe* species. Gardner (1974) indicated that *B. producta*, fed on the corticioid basidiomycete fungus, *Peniophora* sp. (Russulales) and *B. rosea*, has been observed on the corticioid fungus *Merulius* sp. (Polyporales) (Rockerfeller 2012). Surveys of fungi associated with *Brachycybe* species recovered at least 10 genera in the Polyporales, including *Irpex lacteus*, *Trametopsis cervina* and *Junghuhnia nitida* (Kasson et al. 2016, Macias et al. 2019, their supplementary table 1). A study by Macias et al. (2019) used barcode sequencing to identify fungi cultured and isolated from 301 whole macerated individuals. They found that *B. lecontii* associated with fungi in 176 genera and 39 orders. Of these, 12 genera were conserved across different wood substrates and millipede clades (Macias et al. 2019). They found that the most common wood substrates for *B. lecontii* and associated fungi were tulip poplar (*Liriodendron tulipifera*) and oaks (*Quercus* spp.). Through observations in the field, we found that *B. lecontii* almost exclusively fed on crust and bracket-like basidiomycetes. However, Macias et al. (2019) showed that Polyporales, a majority of crust and bracket-like basidiomycetes, only represented 9% of all isolates recovered from macerated individuals and certain polypore taxa were pathogenic to *B. lecontii*. Evidence of fungal feeding on polypores was confirmed by the bowl-shaped depressions (“feeding bowls”) that *B. lecontii* made with its head in the fungal tissue (Fig. 10). How *B. lecontii* fed and made these feeding bowls is uncertain. The millipedes appear to feed exclusively on liquids from fungi because no solid matter was found in the digestive tracts of dissected millipedes, despite observing the millipedes with their heads embedded in the fungal tissue. *Brachycybe lecontii* individuals were observed defecating only liquids, not solids, providing additional support for a liquid-feeding lifestyle. A common reaction of millipedes, when immersed in 70% isopropyl alcohol for preservation, is the regurgitation of their gut contents. Millipedes in the family Xystodesmidae, which feed on decaying leaves, regurgitate an opaque beige liquid that solidifies into a gel (PEM, pers. comm.). However, *B. lecontii*, preserved in this manner, regurgitates a clear liquid, visible in the alcohol by the differential refraction between the two liquids. Blanke and Wesener (2014) used micro-computed tomography and found that *B. lecontii* possesses two large paired gland-like structures extending backwards beyond the two rings posterior to the head. The glands open to the cephalic region near the buccal cavity. These unknown structures of uncertain homology may be salivary glands related to fungal feeding and secrete a chitinase to liquefy fungal tissue or may be associated with the care of eggs and secrete anti-fungal compounds for protection. Alternatively, liquefaction of fungal tissues with the large salivary glands may serve to detoxify otherwise pathogenic fungus and permit communal feeding.

### Feeding Structures

Blanke and Wesener (2014) created a 3D reconstruction of the head and anterior trunk rings of *B. lecontii* and provided a detailed survey of the external and internal morphology. With scanning electron microscopy, we documented additional morphological structures associated with the morphology of the head and feeding. The mouthparts of *B. lecontii* are like those of other colobognath millipedes, having reduced mandibles (not visible externally and with a smooth external surface, without pectinate lamellae and molar plate and with reduced external teeth), split labrum without marginal teeth, distinct gnathochilarial sclerites, reduced inner and outer palps of the gnathochilarium and a closely-appressed labrum and gnathochilarium (Gardner 1974, Marek et al. 2012). The feeding mode may be of a suctorial liquid-feeding nature as Manton (1961) indicated is the mode of some other colobognath millipedes. This feeding mode is supported by the anatomy of the foregut of *B. lecontii*, shown with micro-computed tomography in Blanke and Wesener (2014). The pharynx appears thickened and muscular and the foregut is star-shaped in cross-section (with six inward projecting cristae or folds). The thickened pharynx may serve to make negative pressure in the buccal cavity to draw in fluids and the sieve-like foregut may filter out large fungal particles. A similar star-shaped anatomical appearance and filtering function is in the proventriculus of ants that acts as a sieve for liquid feeding (Eisner and Wilson 1952, Lanan et al. 2016). However, the precise manner in which they extract liquid from fungus is yet unknown, though Lewis (1984) suggested external digestion as a possibility. This would be consistent with bowl-shaped feeding marks that typically include a centrally-located “wet spot” instead of protruding ragged hyphal strands, the latter of which would be consistent with the hyphae being chewed or tugged upon. Feeding by external digestion, perhaps facilitated by liquefaction by saliva, is supported by the presence of spacious cephalic glands found by Blanke and Wesener (2014).

The semicircular structure on the tip of the labrum is distinctive and not observed in any other group of Diplopoda. The numerous pits and setae around the structure suggest that it may be sensory or highly sensitive in nature. The rough texture and raised central ridge of the labrum may, if feeding does not rely on external digestion and, instead, requires mechanical rasping of fungus to extract liquid, aid the millipede in tearing and mashing the fungal hyphae or act as an additional sieve to filter solids from liquid. The split labrum, which externally is rough and raised into a central ridge and continues through to the epipharynx as a cleft, was found in the mouthparts of the colobognath millipede genus *Illacme* (family Siphonorhinidae) that were suggested to be for rasping plant or fungal tissue (Marek et al. 2012, Marek et al. 2016).

### Pinwheeling

Pinwheel aggregations have been described in previous studies and authors speculated about their function. Gardner (1974) referred to the aggregations as “star clusters”, but the term was not adopted in subsequent studies. We chose the term "pinwheeling" as it was descriptive and is itself a more precise behavioural verb, in that "pinwheeling" alludes to the head-in/tail-out pattern of the aggregation and "star clustering" or "clustering" does not.

The pinwheel assembly appears to be a feeding behaviour associated with the presence of fungus, as millipedes kept in containers without fungus did aggregate but did not pinwheel. Persistence of pinwheels (up to 27 days) may correlate to food quality rather than abundance, as some pinwheels were observed dispersing from areas with plentiful fungal growth still present. The aggregation shape may be coincidental, arising as each individual millipede seeks to reach and feed on certain areas of fungal growths or may be functional and aid feeding, such as through pooling of digestive secretions for external digestion, suggested by Lewis (1984) in a study of the closely-related genus *Pseudodesmus* from Sarawak, Malaysia. In addition, the millipedes may form pinwheels to stimulate (by digestive secretions, mechanical stimulation etc.) a fungal growth stage that is more palatable for consumption. The pinwheel arrangement may be multifunctional and evolutionarily co-optimised for feeding and additional functions, such as defence, since fungivory seems to require a long handling time that makes individuals conspicuous and more vulnerable to predation (Speed et al. 2010). For example, when a pinwheel consisting of a mixture of adults and young juveniles was disturbed by us in the field, an ant (*Camponotus* sp.) carried off one of the juvenile millipedes, while solitary wandering adults were ignored by ants (site DAH-2017-0516-01). Pinwheels may be aposematic, signalling their chemical defences to visual predators. The pinwheel aggregation may simply numerically dilute risk or augment the overall aposematic signal, more so than if a solitary individual was viewed by a predator or if the same number of individuals were in a different formation, as by a search image or a supernormal stimulus (Joron 2009). It does not appear that pinwheels serve to enhance chemical defences via pooling the volume of defensive secretions, since when an attack was simulated on an individual millipede in a pinwheel, the other individuals did not react and also did not exude chemicals (also observed by Lewis 1984). Gregarious behaviour, such as pinwheeling, may also facilitate finding of mates, be a result of mate-guarding or function as a lek.

Pinwheels had a female-biased sex ratio (Fig. 13). Lewis (1984) found a similar female-biased composition in his observations of *Pseudodesmus* sp. in Malaysia with 21 females and three males. Pinwheeling does not appear to be associated with the care of eggs or young. Males with eggs were not observed as members of a pinwheel; this may account for the lower proportion of males in pinwheels. In the majority of cases, millipedes have an XY sex determination system (Minelli and Michalik 2015) that would not necessarily be consistent with the female-biased sex ratio in the pinwheels. In addition, Fisher’s principle (1930) predicts that parental investment will be equal amongst the sexes, thereby establishing a 1:1 sex ratio (Fisher 1930). In two cases, pinwheels, consisting of single adult females with several stadium I-II juveniles, were observed. When the females were removed, the juveniles scattered, behaviour also seen when aggregations of solely juveniles were disturbed. Parental or brooding behaviour was not exhibited by the female adults in these pinwheels. Why females are more frequent in pinwheels is uncertain. Often sex-biased aggregations are related to swarming prior to mating, thereby facilitating finding mates or is due to resource or mate-guarding. The latter is consistent with the common behaviour of multiple mating and polygynandry in millipedes (Wojcieszek and Simmons 2012). In most of the occurrence records of *B. lecontii* with a single individual from a single locality, the sex is male. Our observations of aggregations predominately composed of females combined with the higher frequency of lone males, may indicate harem formation and male mate-guarding as is the case in several well-studied species (e.g. gorillas, birds and wetas: Fossey 1974, Ridley and Hill 1987, Gwynne and Jamieson 1998). Harem formation has not been observed in millipedes; however, mate-guarding has been described in the spirostreptidan species *Alloporus uncinatus* and the polydesmidan species *Nyssodesmus python*. In *A. uncinatus*, the male prolongs copulation when other males are present, thereby restricting access to the female (Telford and Dangerfield 1993). In *N. python*, the male rides atop the back of the female after copulation even while the female is walking, for up to five days (Adolph and Geber 1995); however it is uncertain if this behaviour occurs in the presence of other males as is the case in *A. uncinatus*.

### Leg Morphology

The tarsal comb structure of *B. lecontii* is present in related platydesmidan millipedes of the genera *Gosodesmus*, *Andrognathus* and *Dolistenus* (Shorter et al. 2018, Recuero and Rodríguez-Flores 2020). Counts of setae in the tarsal comb generally are more numerous in adult individuals, but no regular pattern is apparent with regards to the predictable increase in the number of setae in the combs according to stadium (Table 4). As parental care of eggs is not known in any of these genera (though it may be present in *Andrognathus*, see Shorter et al. 2018, their figure 1D) and juveniles also have the structure, the tarsal comb structure likely does not aid in parental care of eggs and, instead, is possibly used for a more general function, such as grooming or burrowing. Individual *B. lecontii* have been encountered 10 cm below ground and under rotting wood and detritus (Murakami 1962a) and, therefore, a fossorial function of the tarsal comb seems more likely.

### Colour

The pink (to the human eye) colouration of *B. lecontii* is directly related to high reflectance in the blue (450 nm) and red wavelengths (> 600 nm) of the colour spectrum. These colours additively mix to appear pink in the human visual system. As the brightness (area beneath the spectrum) is low, the pink hue appears darker (Fig. 16). In addition, the saturation or chroma (slope coefficients of the blue and red spectral peaks) is low, so the pink colour appears less vivid. Spectral measurements of reflectance show negligible ultraviolet (UV) reflectance, indicating the colour is likely not derived from UV-reflecting pigments, such as carotenoids that reflect at a peak wavelength of 250 nm. With low UV reflectance, their colouration may lack a radiation-protectant quality in the UV range, an unsurprising finding given our frequent observations of *B. lecontii* occurring beneath decaying logs and in forest understoreys with very low light conditions (Hoffman 1950, Shelley et al. 2005).

The colour of *B. lecontii* has been described as varying geographically within the species from east to west with individuals from Arkansas redder than those from Virginia, which are light pink (Gardner 1974, Shelley et al. 2005). The chemical source and function for the pink colouration of *B. lecontii* is unknown. Other millipedes have six types of pigments including quinones, coproporphyrins, ommochromes, melanins, flavins and tetrapyrroles (Hopkin and Read 1992). Of these pigments, coproporphyrin is the likeliest because it produces a red hue. This pink colouration may be related to predator defence, since chemically defended arthropods have a high apparency to their predators and are aposematic. Notably, dragon millipedes (e.g. the genus *Desmoxytes*), which are chemically defended with hydrogen cyanide, also possess a pink colour (Srisonchai et al. 2018). However, pink is typically not a common aposematic colour in nature and millipedes that have colours that have been directly tested to be aposematic are long wavelength colours (yellow to red) and, in an unusual case, short wavelength bioluminescence (495 nm) (Marek et al. 2011). To address the hypothesis that pink colouration in *B. lecontii* serves as an aposematic signal, controlled predator studies to test association of the millipede’s appearance with its toxicity should be conducted.

## Supplementary Material

25178748-0C24-54B1-904A-A5E7352300E6 10.3897/BDJ.8.e50770.suppl1 Supplementary material 1 *Brachycybe lecontii* pinwheels (Walhalla Co., South Carolina, U.S.A.) Data type movie Brief description This time-lapse movie shows three *Brachycybe lecontii* pinwheels and associated fungus from Issaqueena Falls (Walhalla Co., South Carolina). The 35 frames were photographed 15 minutes apart. File: oo_374858.mp4 https://binary.pensoft.net/file/374858 Marek, P.E.

65FB90F4-222C-5C79-A26E-0276C11E027B 10.3897/BDJ.8.e50770.suppl2 Supplementary material 2 Files with spectra Data type text files with spectrometer data Brief description This archive contains 18 text files with the reflectance (% reflectance by wavelength) measurements underlying figure 16 File: oo_394337.zip https://binary.pensoft.net/file/394337 Wong, V.L.

## Figures and Tables

**Table 1.** Localities of *Brachycybe lecontii* specimens collected for this study, with associated collection information.

**Date**	**Locality code**	**State**	**Coordinates**	**Collectors**	**Habitat**	**Specimens**
29.xii.2015	VLW-2015-001	Virginia	37.0252° -80.7752°	VL Wong, P Marek, J Means, P Shorter	Under *Liriodendron tulipifera* log	MPE00811 - MPE00813
22.iv.2016	MK-2016-010	Arkansas	34.6119° -93.1655°	M Kasson	*Quercus* and *Pinus taeda*	BLC6, BLC8, BLC9
23.v.2016	JCM-2016-033	Alabama	34.0996° -87.3197°	J Means, DA Hennen	On *Magnolia macrophylla* log; *Pinus*, *Acer*, *Fagus grandifolia*, *Quercus*	MPE02306 - MPE02321
25.v.2016	JCM-2016-042	Tennessee	36.2651° -82.2300°	J Means, DA Hennen	Moist litter with dark, loamy soil; *Tsuga*, *Acer*, *Quercus*, *Liriodendron tulipifera*, *Rhododendron*	MPE02064, MPE02066, MPE02071, MPE02076, MPE02080
9.x.2016	JCM-2016-111	Tennessee	36.0486° -83.7487°	J Means, DA Hennen	Dry deciduous litter, some dead branches	MPE02302 - MPE02305
12.v.2017	DAH-2017-0512-02	Tennessee	35.8990° -83.9483°	DA Hennen, J Means, VL Wong	On hardwood log with fungus, in leaf litter; moist (raining); *Quercus*, *Carya*, *Fagus grandifolia*, *Acer*	BLIV0001 - BLIV0007
13.v.2017	DAH-2017-0513-02	Tennessee	36.1017° -87.2853°	DA Hennen, J Means, VL Wong	On hardwood logs, branches with lichen and fungi; in moist deciduous leaf litter, particularly *Fagus grandifolia*	BLCV0001-001 - BLCV0001-015, BLCV0002-001 - BLCV0002-011, BLCV0003-001 - BLCV0003-011, BLIV0008 - BLIV0020
16.v.2017	DAH-2017-0516-01	Arkansas	34.7005° -92.2606°	DA Hennen, J Means, VL Wong	On hardwood log with fungi on underside; *Carpinus caroliniana*, *Carya*, *Acer*, *Asimina triloba*, *Quercus*	BLCV0004-001 - BLCV0004-029, BLCV0005-001 - BLCV0005-007, BLIV0021 - BLIV0045
16.v.2017	DAH-2017-0516-02	Arkansas	36.0376° -93.3412°	DA Hennen, J Means, VL Wong	On hardwood branch, log with fungus, in leaf litter; *Fagus grandifolia*, *Carpinus caroliniana*, *Acer*, *Quercus*	BLIV0046 - BLIV0065
17.v.2017	DAH-2017-0517-01	Arkansas	36.4307° -93.7576°	DA Hennen, J Means, VL Wong	On bark on underside of fallen branch; *Acer*, *Platanus occidentalis*, *Quercus*, *Asimina triloba*, *Juniperus virginiana*	BLIV0066
17.v.2017	DAH-2017-0517-02	Missouri	37.1322° -92.3241°	DA Hennen, J Means, VL Wong	On hardwood branch with fungus, in dry litter; *Quercus*, *Carya*, *Prunus serotina*, *Juniperus virginiana*	BLCV0006-001 - BLCV0006-005, BLIV0067 - BLIV0070
21.v.2017	DAH-2017-0521-02	Virginia	37.2970° -82.3006°	DA Hennen, J Means, VL Wong	On hardwood branch, log, under bark; moist leaf litter; *Quercus*, *Tsuga*, *Rhododendron*, *Acer*, *Liriodendron tulipifera*	BLIV0071 - BLIV0076
9.viii.2017	JCM-2017-047	Tennessee	36.5022° W-82.482	J Means, DA Hennen	Dry *Quercus*, *Acer*, *Carya* forest	BLIV0077 - BLIV0079

**Table 2.** Sexes and life stages of *Brachycybe lecontii* specimens observed at each locality (refer to Table 1 for the locality code). Abbreviation: indet., indeterminate, specimens too young to be sexed by the presence or absence of gonopods.

**Date**	**Locality code**	**Specimen identifier**	**Sex**	**Life Stage**	**Ring Number**
12.v.2017	DAH-2017-0512-02	BLIV0001	Male	Adult	44
		BLIV0002	Female	Adult	51
		BLIV0003	Female	Adult	45
		BLIV0004	Female	Adult	50
		BLIV0005	Female	Adult	52
		BLIV0006	Female	Adult	44
		BLIV0007	Male	Adult	45
13.v.2017	DAH-2017-0513-02	BLCV0001-001	Male	Adult	39
		BLCV0001-002	Female	Juvenile	30
		BLCV0001-003	Female	Juvenile	32
		BLCV0001-004	Female	Juvenile	24
		BLCV0001-005	Female	Juvenile	25
		BLCV0001-006	Indet.	Juvenile	22
		BLCV0001-007	Male	Adult	40
		BLCV0001-008	Indet.	Juvenile	23
		BLCV0001-009	Male	Juvenile	30
		BLCV0001-010	Male	Adult	41
		BLCV0001-011	Male	Adult	44
		BLCV0001-012	Female	Adult	39
		BLCV0001-013	Male	Adult	45
		BLCV0001-014	Female	Juvenile	24
		BLCV0001-015	Indet.	Juvenile	19
13.v.2017	DAH-2017-0513-02	BLCV0002-001	Male	Adult	38
		BLCV0002-002	Female	Juvenile	34
		BLCV0002-003	Female	Adult	56
		BLCV0002-004	Male	Adult	42
		BLCV0002-005	Female	Adult	48
		BLCV0002-006	Female	Adult	52
		BLCV0002-007	Female	Adult	56
		BLCV0002-008	Male	Adult	48
		BLCV0002-009	Female	Adult	41
		BLCV0002-010	Male	Adult	49
		BLCV0002-011	Female	Adult	52
13.v.2017	DAH-2017-0513-02	BLCV0003-001	Female	Adult	52
		BLCV0003-002	Male	Adult	48
		BLCV0003-003	Female	Adult	46
		BLCV0003-004	Female	Adult	42
		BLCV0003-005	Female	Adult	51
		BLCV0003-006	Female	Adult	47
		BLCV0003-007	Female	Adult	41
		BLCV0003-008	Male	Adult	45
		BLCV0003-009	Male	Adult	39
		BLCV0003-010	Female	Adult	50
		BLCV0003-011	Male	Adult	40
13.v.2017	DAH-2017-0513-02	BLIV0008	Female	Adult	48
		BLIV0009	Female	Adult	44
		BLIV0010	Male	Adult	45
		BLIV0011	Female	Adult	43
		BLIV0012	Male	Adult	52
		BLIV0013	Female	Adult	49
		BLIV0014	Female	Adult	53
		BLIV0015	Male	Adult	40
		BLIV0016	Male	Adult	44
		BLIV0017	Female	Adult	46
		BLIV0018	Female	Adult	53
		BLIV0019	Male	Adult	39
		BLIV0020	Female	Adult	45
16.v.2017	DAH-2017-0516-01	BLCV0004-001	Female	Juvenile	32
		BLCV0004-002	Female	Juvenile	32
		BLCV0004-003	Male	Juvenile	35
		BLCV0004-004	Female	Juvenile	32
		BLCV0004-005	Female	Adult	46
		BLCV0004-006	Male	Juvenile	31
		BLCV0004-007	Male	Juvenile	29
		BLCV0004-008	Male	Juvenile	35
		BLCV0004-009	Female	Juvenile	31
		BLCV0004-010	Male	Juvenile	35
		BLCV0004-011	Male	Juvenile	32
		BLCV0004-012	Female	Juvenile	35
		BLCV0004-013	Female	Adult	40
		BLCV0004-014	Female	Juvenile	33
		BLCV0004-015	Male	Juvenile	29
		BLCV0004-016	Female	Juvenile	32
		BLCV0004-017	Indet.	Juvenile	23
		BLCV0004-018	Indet.	Juvenile	23
		BLCV0004-019	Female	Juvenile	27
		BLCV0004-020	Female	Juvenile	26
		BLCV0004-021	Indet.	Juvenile	23
		BLCV0004-022	Male	Juvenile	25
		BLCV0004-023	Female	Juvenile	24
		BLCV0004-024	Female	Juvenile	33
		BLCV0004-025	Male	Adult	35
		BLCV0004-026	Female	Juvenile	34
		BLCV0004-027	Female	Juvenile	33
		BLCV0004-028	Indet.	Juvenile	19
		BLCV0004-029	Indet.	Juvenile	17
		BLCV0005-001	Female	Adult	39
		BLCV0005-002	Female	Juvenile	28
		BLCV0005-003	Indet.	Juvenile	23
		BLCV0005-004	Female	Juvenile	27
		BLCV0005-005	Female	Juvenile	34
		BLCV0005-006	Male	Juvenile	24
		BLCV0005-007	Female	Juvenile	34
		BLIV0021	Indet.	Juvenile	18
		BLIV0022	Female	Juvenile	28
		BLIV0023	Indet.	Juvenile	23
		BLIV0024	Indet.	Juvenile	24
		BLIV0025	Female	Juvenile	31
		BLIV0026	Female	Juvenile	32
		BLIV0027	Male	Adult	38
		BLIV0028	Male	Adult	46
		BLIV0029	Male	Adult	37
		BLIV0030	Male	Adult	39
		BLIV0031	Male	Adult	36
		BLIV0032	Male	Juvenile	29
		BLIV0033	Male	Adult	41
		BLIV0034	Male	Juvenile	32
		BLIV0035	Female	Juvenile	31
		BLIV0036	Male	Juvenile	30
		BLIV0037	Male	Adult	–
		BLIV0038	Male	Adult	38
		BLIV0039	Female	Adult	48
		BLIV0040	Female	Adult	47
		BLIV0041	Female	Adult	48
		BLIV0042	Male	Adult	39
		BLIV0043	Male	Adult	40
		BLIV0044	Female	Adult	41
		BLIV0045	Female	Juvenile	33
16.v.2017	DAH-2017-0516-02	BLIV0046	Female	Adult	50
		BLIV0047	Female	Adult	47
		BLIV0048	Female	Juvenile	30
		BLIV0049	Male	Adult	44
		BLIV0050	Male	Adult	41
		BLIV0051	Male	Adult	48
		BLIV0052	Male	Adult	42
		BLIV0053	Female	Adult	50
		BLIV0054	Female	Adult	51
		BLIV0055	Female	Adult	47
		BLIV0056	Female	Adult	47
		BLIV0057	Female	Adult	42
		BLIV0058	Female	Adult	40
		BLIV0059	Female	Adult	46
		BLIV0060	Female	Adult	53
		BLIV0061	Female	Adult	39
		BLIV0062	Female	Adult	48
		BLIV0063	Female	Adult	45
		BLIV0064	Female	Adult	41
		BLIV0065	Female	Adult	49
17.v.2017	DAH-2017-0517-01	BLIV0066	Female	Adult	51
17.v.2017	DAH-2017-0517-02	BLCV0006-001	Female	Adult	50
		BLCV0006-002	Female	Juvenile	28
		BLCV0006-003	Female	Juvenile	29
		BLCV0006-004	Indet.	Juvenile	20
		BLCV0006-005	Indet.	Juvenile	18
21.v.2017	DAH-2017-0521-02	BLIV0067	Male	Adult	49
		BLIV0068	Female	Juvenile	36
		BLIV0069	Female	Adult	47
		BLIV0070	Female	Juvenile	49
		BLIV0071	Female	Adult	51
		BLIV0072	Female	Adult	50
		BLIV0073	Female	Adult	52
		BLIV0074	Female	Adult	42
		BLIV0075	Female	Adult	58
		BLIV0076	Female	Adult	51

**Table 3.** Proximity of *Brachycybe lecontii* individuals to fungus, observed during Spring 2017. Millipedes were engaged in feeding on fungal mats at all but two localities. (^1^Fungus present on the log or branch from where the individual or pinwheel was encountered or within 10 cm of the individual or pinwheel. ^2^No fungus present on the log or branch where the individual or pinwheel was encountered or not within 10 cm of the individual or pinwheel. The majority of individuals that were not found on or near fungus were found in leaf litter.).

**Locality**	**On fungus**	**Fungus nearby^1^**	**Fungus not nearby^2^**
DAH-2017-0512-02	0	7(BLIV001 - BLIV007)	0
DAH-2017-0513-02	39(BLCV0001 - BLCV0003, BLIV0019 - BLIV0020)	3(BLIV0008, BLIV0009 - BLIV0011)	7(BLIV0010, BLIV0013 -BLIV0018,)
DAH-2017-0516-01	41(BLCV0004, BLCV0005, BLCV0007, BLCV0008, BLIV0041 - BLIV0045)	7(BLIV0021-BLIV0026, BLIV0037)	11(BLIV0027 - BLIV0036, BLIV0038)
DAH-2017-0516-02	6(BLCV0009, BLIV0048 - BLIV0048)	17(BLIV0049-0065)	0
DAH-2017-0517-01	1(BLIV0066)	0	0
DAH-2017-0517-02	0	5(BLCV0006)	4(BLIV0067 - BLIV0070)
DAH-2017-0521-02	6(BLIV0071 - BLIV0076)	0	0

**Table 4.** Counts of setae in combs on the tarsus (and tibia, in parentheses) on the six anterior-most leg pairs (LP 1–6) of *Brachycybe lecontii*. Some legs of specimens that were mounted on SEM stubs were missing and, therefore, the setae count could not be obtained. Leg pairs that are missing data or undeveloped are denoted with “–”.

**Specimen identifier**	**Sex**	**No. rings**	**LP 1**	**LP 2**	**LP 3**	**LP 4**	**LP 5**	**LP 6**
BLIV0020	Female	45	3 (2)	8 (3)	12 (6)	12 (0)	10 (0)	4 (0)
BLIV0028	Male	46	7 (4)	15 (4)	15 (5)	13 (4)	4 (0)	3 (0)
BLIV0029	Male	37	6 (4)	9 (4)	10 (2)	6 (0)	3 (0)	2 (0)
BLIV0031	Male	36	5 (3)	9 (3)	9 (4)	8 (0)	4 (0)	0 (0)
BLIV0032	Male	29	5 (3)	7 (3)	10 (2)	7 (0)	3 (0)	1 (0)
BLIV0077	Indet.	16	3 (2)	5 (2)	5 (2)	–	–	–
BLIV0078	Indet.	14	3 (1)	4 (2)	5 (1)	1 (0)	–	–
BLIV0079	Indet.	20	5 (1)	7 (2)	6 (0)	2 (1)	–	–
MPE02315	Indet.	22	–	6 (3)	8 (4)	6 (0)	4 (0)	–
MPE02316	Female	25	4 (0)	9 (2)	8 (3)	–	3 (0)	–
MPE02378	Indet.	7	3 (1)	4 (2)	2 (0)	–	–	–
